# Production and evaluation of a novel GABA-producing almond yogurt: dietary modulation of stress

**DOI:** 10.3389/fvets.2026.1949462

**Published:** 2026-09-21

**Authors:** Murali Anagha, Roshni Ramachandran, Gangaraju Divyashri, T.P. Krishna Murthy

**Affiliations:** 1 Department of Biotechnology, M S Ramaiah Institute of Technology, Bengaluru, Karnataka, India; 2 Bioproducts Research, Iom Bioworks Pvt. Ltd, Centre for Cellular and Molecular Platforms (C-CAMP), Bengaluru, Karnataka, India

**Keywords:** almond yogurt, *Enterococcus faecalis* RIT BMS15-3, functional food, gamma-aminobutyric acid (GABA), psychobiotics

## Abstract

Gamma-aminobutyric acid (GABA) is an inhibitory neurotransmitter recognized for its antihypertensive, anxiolytic, neuroprotective, and cognitive-enhancing properties. Functional foods enriched with naturally produced GABA have emerged as promising dietary interventions to modulate the microbiota-gut-brain axis and promote mental well-being. In the current study, a novel GABA-enriched plant-based almond yogurt was developed using the psychobiotic strain *Enterococcus faecalis* RIT BM_S15-3_, a human breast milk-derived isolate, as the starter culture. To the best of our knowledge, this is the first report highlighting the use of a human breast milk-derived psychobiotic *Enterococcus faecalis* RIT BM_S15-3_ to produce GABA-enriched almond yogurt. The fermentation parameters were systematically optimized to obtain maximum GABA enrichment while ensuring microbial viability and product quality. Unlike previous studies that primarily optimize plant-based yogurts for texture or antioxidant properties, this study focused on maximizing microbial GABA biosynthesis by optimizing L-monosodium glutamate (L-MSG) concentration, fermentation temperature, and fermentation time. The confirmatory experiment resulted in a GABA concentration of 29 mM in the final yogurt developed almond yogurt exhibited high antioxidant activity in DPPH and ABTS assays, with total phenolic and flavonoid contents showing higher activity than those of commercially available dairy and non-dairy yogurt. We optimized the fermentation conditions to improve GABA yield, including L-monosodium glutamate (L-MSG) concentration, fermentation time, and fermentation temperature. The results indicated a GABA yield of 29 mM in the yogurt after optimization. The almond yogurt showed a suitable water-holding capacity, viscosity, and favorable sensory acceptability (overall score: 7.52), as well as functional stability during refrigerated storage. A 21-day storage study showed sustained GABA retention together with maintenance of viable probiotic populations, indicating good functional stability of the developed product. This study emphasizes the feasibility of developing a naturally GABA-enriched, probiotic almond yogurt as a potential next-generation functional food with prospective applications in microbiota-gut-brain axis modulation. Although the observed functional characteristics suggest promise for supporting neurological health, further *in vivo* and clinical studies are required to establish anti-anxiety and neuroprotective efficacy.

## Graphical abstract

**Figure:**
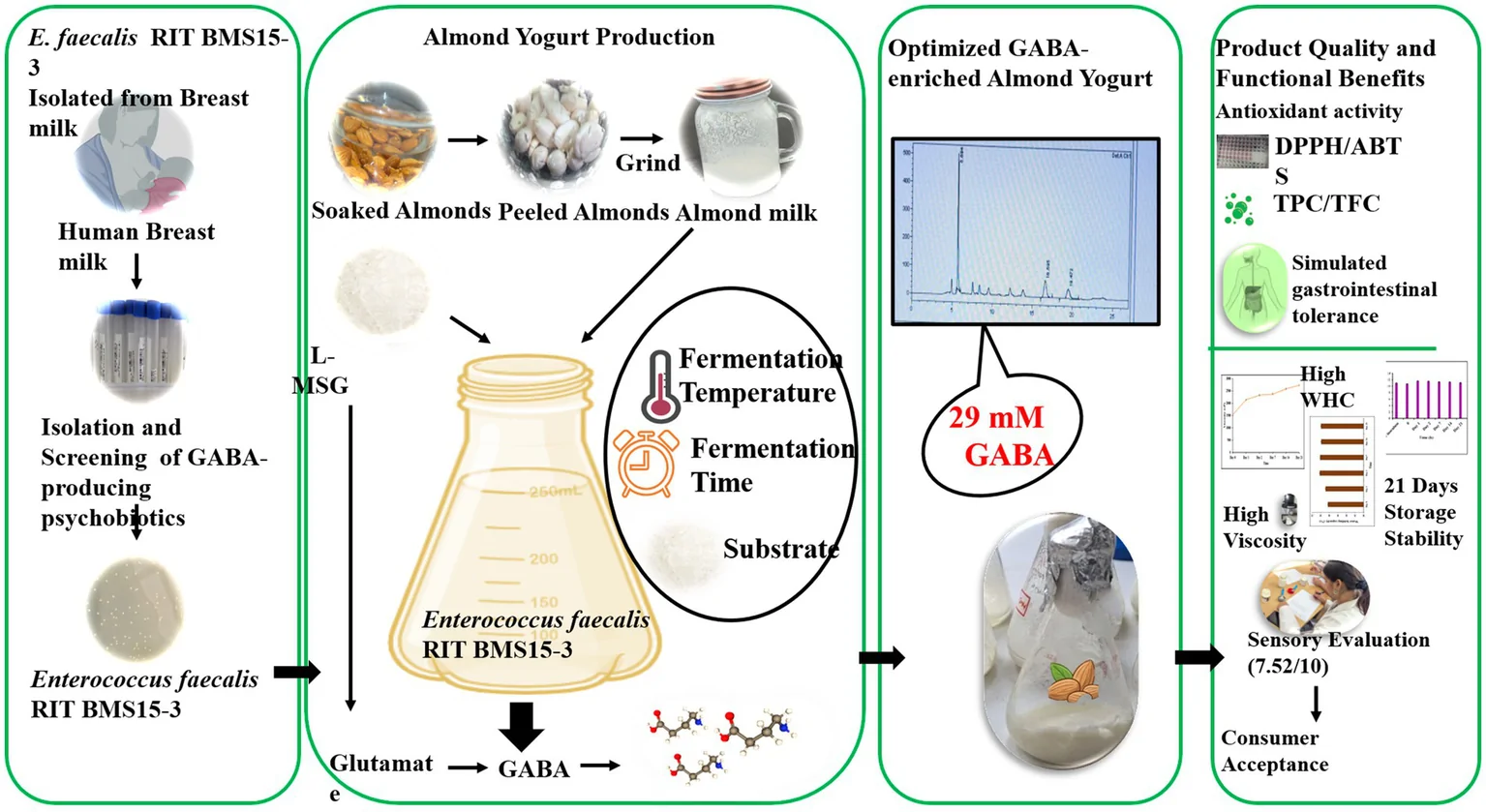

## 1 Introduction

Health has been a predominant focus in the everyday life of people at all times. Studies prove that globally there is an increase in the prevalence of mental health disorders such as depression, anxiety, and stress despite decades of pharmacological intervention (1). According to the World Health Organization (WHO), approximately 970 million people worldwide were living with a mental disorder in 2019, highlighting the substantial global burden of mental health conditions (2). Beyond their psychological burden, these disorders are increasingly recognized as systemic conditions characterized by chronic physiological stress, dysregulation of the hypothalamic–pituitary–adrenal (HPA) axis, oxidative stress, and alterations in the gut microbiota. Although pharmacological treatments such as selective serotonin reuptake inhibitors (SSRIs), benzodiazepines, and gabapentinoids (gabapentin and pregabalin) are commonly used in the management of anxiety and related disorders, their use may be associated with class-specific adverse effects. SSRIs may cause adverse effects including gastrointestinal disturbances, sexual dysfunction, and sleep-related symptoms (3). Benzodiazepines may be associated with sedation, cognitive and psychomotor impairment, tolerance, dependence, and withdrawal symptoms (4). Gabapentin and pregabalin may cause adverse effects including dizziness and somnolence, among other central nervous system-related effects (5). Notably, many of these side effects mirror the very metabolic and dietary dysregulations that seem to worsen mood disorders. This has directed increasing scientific attention towards preventive, diet-based strategies, especially those targeting the gut-brain axis, as more sustainable long-term approaches to mental well-being. Recent advances in gut microbiome research show that dietary interventions that modulate microbial metabolism may influence neurochemical signaling through the microbiota-gut-brain axis. Among these neuroactive metabolites, GABA has attracted considerable attention as certain probiotic microorganisms can synthesize it during fermentation, offering opportunities to develop functional foods with potential psychobiotic benefits.

Functional foods represent one of the most practical frameworks for dietary intervention. According to the European Commission Concerted Action on Functional Food Science in Europe (FUFOSE), functional foods are those demonstrated to beneficially affect one or more target functions in the body, beyond basic nutrition, in ways relevant to improved health or reduced disease risk. Yogurts are among the most extensively studied; they are included in every national dietary guideline (6) and are broadly tolerated across age groups. Their fermented nature confers a wide range of functional benefits, including improved gut integrity, enhanced immune response, and better lactose digestibility (7–9). The probiotic content of yogurt has also been associated with beneficial physiological outcomes in several clinical studies, likely through modulation of the gut-brain axis. Despite this strong profile, conventional dairy-based yogurt remains inaccessible to a substantial portion of the global population, including those with lactose intolerance, casein allergy, or plant-based dietary preferences (10). Global lactose intolerance rates exceed 65% in adults, and the commercial demand for non-dairy fermented products has grown considerably across both Western and Eastern markets (11). This gap has driven growing interest in plant-based yogurt alternatives that preserve the functional attributes of traditional dairy yogurt while broadening inclusivity.

Almond milk has become one of the most widely adopted plant-based dairy substitutes, and there are nutritionally valid reasons for the same. Almonds are rich in monounsaturated fatty acids, plant proteins, dietary fiber, magnesium, manganese, and vitamin E, a composition that supports cardiovascular function, glycemic stability, and antioxidant defense (12). Beyond their macronutrient profile, almonds consist of prebiotic fibers and bioactive polyphenols that selectively promote the metabolic activity and the survival of beneficial gut bacteria, making almond milk not only a dairy substitute, but also a potentially favorable fermentation substrate for the development of functional food (13, 14). Almond yogurt, produced by fermenting almond milk with lactic acid bacteria (LAB), retains most of the native nutritional attributes of almonds while acquiring the added benefits of live probiotic bacteria, organic acids, and fermentation-derived bioactive compounds. The end product is inherently free of lactose, low in saturated fat, and casein-free, the characteristics that make it suitable for lactose-intolerant, vegan, and allergy-prone consumers (15). Fermentation also enhances the bio accessibility of nutrients derived from almonds through partial breakdown of antinutritional factors such as tannins and phytase, further increasing its functional value (16). Despite these benefits, most commercially available fermented plant-based yogurts lack standardized probiotic strains and offer limited functional enrichment, representing a clear formulation opportunity.

Gamma-aminobutyric acid (GABA) is the major inhibitory neurotransmitter in the mammalian central nervous system. It acts by binding to GABA-A and GABA-B receptors to diminish neuronal excitability, thereby modulating anxiety, improving sleep quality, and attenuating the physiological response to stress (17). Clinically, deficiency of GABA has been connected to anxiety disorders, depression, epilepsy, and insomnia. These conditions are currently managed mostly through synthetic GABA-modulatory drugs, whose long-term use carries well-established risks (3). Dietary GABA supplementation has therefore attracted considerable research interest. Certain LAB are capable of producing GABA endogenously through the action of glutamate decarboxylase (GAD), an enzyme that catalyzes the irreversible decarboxylation of L-glutamate to GABA (18). This conversion occurs efficiently during fermentation, and GABA production is influenced by strain-specific GAD activity, substrate (glutamate) concentration, pH, fermentation time, and temperature. GABA, consumed orally, especially when produced through microbial fermentation in the gut or ingested through fermented foods, has been reported to modulate the hypothalamus-pituitary–adrenal (HPA) axis and vagal signaling, suggesting peripheral GABA may exert central effects through bidirectional gut-brain communication pathways. Food-grade GABA enrichment through LAB fermentation is thus a biologically feasible and commercially scalable strategy for developing neuronutritionally relevant functional foods.

Although dietary GABA displays limited permeability across the blood–brain barrier, accumulating evidence suggests that GABA-producing probiotics may exert beneficial physiological effects through indirect mechanisms, including microbiota-gut-brain axis communication. These mechanisms include modulating vagal nerve signaling, regulating immune and inflammatory pathways, producing neuroactive microbial metabolites, and normalizing hypothalamic–pituitary–adrenal (HPA) axis activity. Consequently, GABA-enriched fermented foods are increasingly being investigated as complementary nutritional strategies for reducing stress and anxiety while promoting neurological health.

*Enterococcus faecalis* RIT BM_S15-3_ has been reported as a high-yield GABA-producing probiotic strain (19). The strain demonstrated efficient bioconversion of L-glutamate to GABA under fermentation conditions typical of food-grade matrices. *E. faecalis* RIT BM_S15-3_, as characterized, exhibits strong probiotic attributes such as acid and bile tolerance, antimicrobial activity against common pathogens, hemolysis test, antibiogram, alongside demonstrable GABA biosynthetic ability. Incorporating the strain into an almond milk matrix presents an opportunity to develop a functional food product that is simultaneously dairy-free, GABA-containing, and probiotic. Previous studies have demonstrated the feasibility of GABA enrichment in plant-based fermented products, which involves almond-based beverages fermented with the GABA-producing *Lactiplantibacillus plantarum* L42g (20). Other plant-based GABA-fermented products have also been reported, suggesting that GABA-enrichment of non-dairy fermented foods is an emerging area rather than an unexplored one. However, the application of a human breast milk-derived psychobiotic *E. faecalis* strain in an almond yogurt remains insufficiently explored (13, 15). Therefore, the current study aimed to develop and characterize a novel GABA-enriched almond yogurt fermented with *E. faecalis* RIT BM_S15-3_, evaluating its functional attributes, including physicochemical properties, GABA content, probiotic viability, antioxidant activity, safety analysis, sensory acceptability, and GABA stability during storage.

Even though numerous studies have reported GABA production in dairy yogurt (13, 15) or soy-based (21) fermented products using conventional lactic acid bacteria, GABA-enriched almond-based fermented products remain relatively limited, particularly those developed using psychobiotic strains isolated from human breast milk. For example, Buatong et al. (20) demonstrated GABA production and antioxidant activity in fermented almond-based beverages using *Lactiplantibacillus plantarum* L42g. Thus, the novelty of the present study is not the first production of a GABA-enriched almond-based fermented product, but rather the application of the human breast milk-derived psychobiotic *E. faecalis* RIT BM_S15-3_ in an almond yogurt. Further, previous investigations have largely focused on different fermentation and product-quality parameters, while this study applied a systematic optimization strategy specifically targeting microbial GABA biosynthesis. The optimization framework used here was guided by the multifactorial approach reported by Zhu et al. (18), while extending this framework to an almond-based yogurt matrix and a distinct GABA-producing strain. Reports on the simultaneous retention of GABA and probiotic viability during storage are comparatively limited for plant-based fermented products.

Therefore, to the best of our knowledge, this study is the first to apply the human breast milk-derived psychobiotic strain *E. faecalis* RIT BM_S15-3_ for developing a GABA-enriched almond yogurt. This novelty claim is limited to the strain-matrix combination, rather than to GABA-enriched plant-based fermentation in general. The study systematically optimized critical fermentation variables to maximize GABA production. It comprehensively evaluated the resulting product for physicochemical characteristics, probiotic viability, antioxidant activity, safety, and sensory acceptability, with simultaneous 21-day monitoring of both GABA stability and viable probiotic populations during refrigerated storage. These investigations provide important evidence supporting the feasibility of formulating a stable, plant-based psychobiotic food with potential relevance to microbiota-gut-brain axis modulation.

## 2 Materials and methods

### 2.1 Materials

#### 2.1.1 Chemicals and reagents

Glucose, starch, peptone, and cysteine hydrochloride were procured from Sigma-Aldrich (USA). De Man Rogosa and Sharpe (MRS) broth granulated, MRS agar, Monosodium glutamate (MSG), Nutrient broth, Mueller-Hinton (MH) broth, and MH agar were procured from Hi Media, India. 2–2.5 kg of almonds (California) were ordered online. Acetonitrile and methanol (HPLC grade) were procured from SDFine India. Standard GABA was procured from Avra Synthesis Private Ltd., India. All the chemicals and solvents used are of analytical grade.

#### 2.1.2 Microorganisms

*Enterococcus faecalis* RIT BM_S15-3_ was taken from the microbiology lab, Department of Biotechnology, Ramaiah Institute of Technology, Bangalore. It was isolated from healthy human breast milk samples (19). The strain was revived from glycerol stock stored at −80 °C in MRS broth at 37 °C for 24–48 h. It was subcultured twice. The microbial load of the inoculum was initially estimated using a spectrophotometer and again verified through microbial spread plating. Before incorporation into almond milk, the cells were harvested by centrifugation, washed twice with sterile distilled water, and the pellet was collected.

### 2.2 Methods

#### 2.2.1 Preparation of GABA-enriched almond yogurt

Almonds (California, Wonderland Foods) were procured from Bangalore. The milk was prepared according to the protocol by (69) with slight modifications and is given in Table 1, which describes the preparation of the almond milk base adapted from (69); the subsequent formulation and fermentation conditions specific to the GABA-enriched almond yogurt developed in the present study are described below. Almond milk, along with 1.5 g/L L-MSG as a substrate, was homogenized and pasteurized at 95 °C for 15 min, and then cooled to 43 °C. Fermented almond yogurt was prepared by incorporating 10% (v/v) of the probiotic strain *E. faecalis* RIT BM_S15-3_, which was first inoculated into MRS medium and incubated for 24 h. The pellet was washed 2–3 times and inoculated into sterilized almond milk. This was then incubated at 37 °C for 24–48 h. A few parameters were tested to optimize GABA yield in almond yogurt, and the yogurt was stored at 4 °C for 21 days to assess storage stability based on commercial yogurt shelf life and storage conditions in the market. Further, viscosity, water holding capacity (WHC), sensory evaluation, and GABA stability tests were carried out. The factors considered were as follows: fermentation time, fermentation temperature, and L-MSG concentration.

**Table 1:** Preparation of almond milk used for GABA-enriched almond yogurt [Adapted from (69)].

Preparation step
Procurement of almonds
Soak almonds in water for 8–10 h/overnight
Peel the skin of soaked almonds to remove trypsin, which is a protein inhibitor
Soak again for 8–10 h
Grinding the soaked almonds (100 g) in a mixer with 100 mL of warm water
Transfer the prepared almond paste to a beaker and filter to remove any foreign particles. Pasteurize for 15 min at 95 °C
Cool the pasteurized milk to room temperature

The almond milk preparation is followed by the L-MSG supplementation, 95 °C/15 min heat treatment, 10% (v/v) inoculation, fermentation, and subsequent storage conditions were specific to the GBA-enriched almond yogurt developed in the present study and are described in Section 2.2.1.

#### 2.2.2 Determination of viable bacterial count in almond yogurt

A 1 g sample of yogurt was mixed with 9 mL of sterile distilled water, and serial dilution was carried out. The MRS agar plates were then spread with 100 μL of the respective diluents and incubated at 37 °C for 24–48 h. Single colonies were counted, and the unit of viable cell count was expressed as CFU/mL. The viable cells were calculated using the Equation 1.


$$\mathrm{CFU}/\mathrm{mL}=\frac{\mathrm{No}.\text{of colonies formed}\times \text{dilution factor}}{1\;\mathrm{mL}\;\text{of sample}}$$ (1)

#### 2.2.3 Tolerance to simulated gastrointestinal fluid (SGF)

The probiotic potential of *E.faecalis* RIT BM_S15-3_ was assessed by inoculating the strain under simulated gastrointestinal conditions (22). 1 mL of almond yogurt sample was mixed with 9 mL of salivary amylase (100 U/mL). Salivary amylase was prepared in 0.1 M sodium acetate buffer at pH 5.6, and the mixture was incubated at 37 °C for 2 min. Further, 1 mL of this sample from the amylase mix was transferred to 9 mL of simulated gastric fluid (SGF) composed of starch (3 g/L), pectin (2 g/L), mucin (1 g/L), glucose (0.1 g/L) yeast extract (3 g/L), peptone (1 g/L) and cysteine HCl (0.5 g/L) and incubated at 37 °C for 3 h. Cell counts were measured at different time intervals using the spread plate technique after serial dilution in sterile water. Subsequently, 1 mL of gastric mixture was transferred to simulated intestinal fluid (SIF) composed of SGF along with 0.5% oxgall and 2.5 g/L of sodium bicarbonate. pH of the mixture was adjusted to 6.5, and it was incubated for 16 h at 37 °C. Samples were collected at different time intervals, and viable cell count was determined by spread plating on MRS agar plates after serial dilution in sterile water (19). The plates were incubated at 37 °C for 24–48 h

#### 2.2.4 Tolerance to simulated intestinal enzymes (trypsin, amylase, lipase)

The intestinal tolerance of *E. faecalis* RIT BM_S15-3_ was further evaluated by exposing the cells to pancreatic enzymes such as trypsin, amylase, and lipase (23). The overnight culture of the strain from almond yogurt was centrifuged at 8,000 rpm for 15 min at 4 °C. The cell pellet was washed twice with sterile phosphate-buffered saline (PBS, pH 7.2) and resuspended to obtain an approximate cell density of 10^8^ CFU/ML. 10% (v/v) of the bacterial suspension was mixed with SIF composed of pancreatic enzymes and incubated at 37 °C for 3 h. Control without enzymes was maintained under identical conditions. Samples were withdrawn at predetermined time intervals (0 h and 3 h), serially diluted in sterile saline, and plated on MRS agar. These plates were then incubated at 37 °C for 24–48 h, and viable cell counts were determined. Survival under the simulated intestinal enzymatic conditions was expressed as log CFU/mL and calculated by comparing viable counts before and after enzymatic exposure as percentage viability (19) using Equation 2.


$$\text{Survival}(\%)=\frac{\mathrm{CFU}\;\text{after enzyme treatment}}{\mathrm{CFU}\;\text{of Control}}\times 100$$ (2)

#### 2.2.5 Estimation of pH and Total Titratable acidity (TTA)

The change in pH of almond yogurt was observed for 21 days using a pH meter (Systronics Digital pH meter 335) at room temperature after calibrating it with different pH buffers (pH: 4.01, 7.00, and 9.21). TTA of the almond yogurt was measured according to the modified method of Al Zahrani et al. (24). 1 mL of the yogurt sample was added to 9 mL of distilled water, and 3 drops of phenolphthalein indicator were added. This solution was titrated against 0.1 N NaOH until a light pink color was observed. TTA (%) was then calculated using Equation 3.


$$\;\;\;\;\mathrm{TTA}(\%)=\frac{\left(\mathrm{Vx}90\mathrm{xN}\right)}{\left(W\right)}\times 100$$ (3)

Where V represents the volume of NaOH used, N is the normality of NaOH, W is the weight of the sample taken, and 90 is the equivalent weight of lactic acid.

#### 2.2.6 Estimation of antioxidant activity (DPPH, ABTS)

Antioxidant activity of the almond yogurt was carried out using the modified 1,1-diphenyl-2-picryl-hydrazyl (DPPH) assay and 2,2’-azinobis (3-ethylbenzothiazoline-6-sulfonic acid (ABTS) assay (25). The almond yogurt sample was prepared before performing the DPPH assay to extract antioxidant compounds from the yogurt matrix, eliminate turbidity and interfering substances, and ensure compatibility with assay solvents for accurate spectrophotometric quantification. After extracting 1 g of sample with 9 mL of methanol, the mixture was vortexed and sonicated for 10 min. Then, 1 mL of 10% TCA was added and kept on ice for 10 min. It was then centrifuged at 12,000 rpm for 15 min at 4 °C. The clear middle layer was collected, and lipids were removed by adding an equal amount of n-hexane, then discarding the hexane layer and filtering using a 0.45 μm PTFE membrane filter (26). To perform the DPPH assay, 0.2 mL of sample was mixed with 1.8 mL of 0.2 mM DPPH solution. The mixture was then incubated for 30 min at room temperature in the dark. Absorbance was measured at 517 nm using a multi-mode plate reader.

Likewise, for ABTS, sample preparation was performed to extract soluble antioxidant compounds from the yogurt matrix, to remove turbidity and other interfering components, and to ensure a homogeneous system for accurate spectrophotometric measurement of ABTS radical scavenging activity. 1 g of almond yogurt was mixed with 9 mL of distilled water or phosphate buffer and vortexed for 2 min, then sonicated for 10 min. Protein precipitation was carried out by sequentially adding 0.5 mL of Carrez 1 (15% potassium ferrocyanide) and Carrez 2 (30% zinc acetate) reagents, then incubating for 10 min. It was then centrifuged at 1,2000 rpm for 15 min at 4 °C. The cell supernatant was filtered using a 0.45 μm PTFE membrane filter (26). The ABTS solution was prepared by mixing 7 mM ABTS with potassium persulfate and kept in the dark at room temperature overnight. The absorbance (734 nm) of the solution was adjusted to 0.7 ± 0.02 using ethanol. For the ABTS assay, 0.2 mL of the sample was mixed with 2.8 mL of the ABTS solution. This mixture was then incubated at room temperature for 10 min, and the absorbance was measured at 734 nm using a multi-mode plate reader. The reference standard used was ascorbic acid. Antioxidant activity was measured using Equation 4 and expressed as % inhibition of free radicals.


$$\text{Antioxidant activity}(\%)=\frac{\left(\mathrm{Ac}-\mathrm{As}\right)}{\mathrm{Ac}}\times 100$$ (4)

Ac, Absorbance of the control (DPPH solution), and As, Absorbance of the sample.

#### 2.2.7 Estimation of Total phenolic content (TPC)

The total phenolic content of the almond yogurt was quantified using the Folin–Ciocalteu (FC) colorimetric method, following the procedure described by (70) with minor modifications. 0.5 mL of the sample was added to 0.5 mL of distilled water, followed by the addition of 1 mL of FC reagent diluted 1:1. This mixture was incubated at room temperature for 5 min. Subsequently, 0.5 mL of 7.5% (w/v) sodium carbonate (Na_2_CO_3_) was added, and the reaction mixture was incubated in the dark at room temperature for 30 min. The absorbance was then recorded at 765 nm using a multi-mode plate reader. Gallic acid was used to generate the calibration curve, and the results were expressed as micrograms of gallic acid equivalents per milliliter (μg GAE/mL).

#### 2.2.8 Determination of Total flavonoid content (TFC)

Total flavonoids in the almond yogurt were detected using the aluminum chloride colorimetric method as described by (70). Briefly, a 0.5 mL sample was mixed with 0.3 mL NaNO_3_ and incubated at RT for 10 min. Then, 0.3 mL 5% (w/v) AlCl_3_ was added and incubated at RT for another 5 min. To terminate the reaction, 1 M NaOH was added, and then the absorbance was measured at 510 nm using a UV–vis spectrophotometer. A calibration curve was generated using quercetin standard solution, and TFC was expressed as micrograms of quercetin equivalents per milliliter (μg QE/mL).

#### 2.2.9 One-factor-at-a-time evaluation and confirmatory experiment for GABA production in almond yogurt

We used 9 mL of almond milk prepared as mentioned in Table 1 for the GABA production experiments. For each experiment, the almond milk was supplemented with L-MSG as a substrate at the concentration specified for the respective treatment, homogenized, pasteurized at 95 °C for 15 min, and cooled down to 43 °C. Further, 10% (v/v) *E. faecalis* RIT BM_S15-3_ was added to this mixture and incubated under the specified fermentation conditions. The effect of individual fermentation parameters on GABA production was evaluated using a one-factor-at-a-time (OFAT) approach, in which fermentation time (8–48 h), temperature (25, 30, 35, 37, 40, 45, and 50 °C), and L-MSG concentration (0.5, 1.0, 1.5, 2.0, 2.5, 3.0, 3.5, and 4.0 g/L) were varied individually, whereas the other experimental conditions were maintained at the respective fixed levels used for each experiment.

Based on the highest GABA production obtained in the individual OFAT experiments, 48 h fermentation time, 37 °C fermentation temperature, and 3 g/L L-MSG concentration were selected for a subsequent confirmatory experiment. The selected conditions were combined, and almond milk containing 3 g/L L-MSG was inoculated with 10%(v/v) *E. faecalis* RIT BM_S15-3_ and fermented at 37 °C for 48 h. The confirmatory experiment was performed to determine the GABA concentration under the combined selected conditions.

Following fermentation, samples were centrifuged to separate the cells, and the cell-free supernatants were collected for GABA quantification. The GABA concentration was expressed in mM.

Different factors were evaluated to optimize GABA yield in almond yogurt; subsequently, the yogurt was stored at 4 °C for 21 days continuously to evaluate its storage stability based on the shelf life of commercially available yogurt in the market. Viscosity, water-holding capacity (WHC), and GABA stability tests were then carried out. The factors evaluated were fermentation time (8 to 48 h), fermentation temperature (25, 30, 35, 37, 40, 45, and 50 °C), and L-MSG concentration (0.5 to 4.0 g/L).

#### 2.2.10 Determination of GABA content

The concentration of GABA was quantified using reverse-phase high-performance liquid chromatography (HPLC). A 10 μL aliquot of the cell-free supernatant was derivatized with 20 μL OPA-MCE reagent and 100 μL sodium borate buffer (0.4 M, pH 10.4) at 30 ± 2 °C for 5 min (71). The mixture was filtered through a 0.22 μm syringe filter (Millipore, MA, United States) and analyzed using a Shimadzu LC-20 AD HPLC system with an SPD-20A UV detector. A 20 μL sample was injected into a Thermo Fisher analytical column. The mobile phase comprised triethylamine (200 μL) and sodium acetate (1.64 g) in 1,000 mL of 20% (v/v) acetonitrile. The flow rate was 0.5 mL/min, and detection was performed at 338 nm at 30 ± 2 °C (27).

#### 2.2.11 WHC test

The WHC test analyses the compactness and texture of the almond yogurt. A higher percentage indicates better water retention. The samples were stirred to homogenize and centrifuged for 15 min at 8,000 rpm at 4 °C. WHC (% w/w) was then evaluated as the weight change after water removal, according to Equation 5 (18).


WHC=100(Y–W)/Y (5)

Where *Y* is the weight of the almond yogurt sample, and *W* is the weight of water expelled.

#### 2.2.12 Rheological studies in the yogurt

The viscosity of almond yogurt was calculated at different fermentation time periods using a Viscometer (Brookfield, United States). The samples were previously incubated at 4 °C and then brought to room temperature. It was then fitted with an LV-3 spindle at 30 rpm.

#### 2.2.13 Safety analysis of *Enterococcus faecalis* RIT BM_S15-3_

Because the isolate sourced from human breast milk belongs to the *Enterococcus* genus, safety assessment was performed using whole-genome sequencing (WGS). Genomic DNA of the isolate was extracted and subjected to whole-genome sequencing using an Illumina paired-end (read length: 150 × 2 bp) sequencing platform (NovaSeq 6000 sequencer). The raw sequencing reads generated were used for genome assembly, annotation, and genome-based safety analysis. In the present study, WGS data were used solely to confirm genomic safety.

#### 2.2.14 Sensory evaluation

The sensory evaluation of almond yogurt assessed odor, taste, appearance, acidity, texture, and overall acceptance by 25 professional panelists, with scores from 1 to 9. Control groups were dairy and non-dairy yogurts purchased from the market. The scoring rules used for the evaluation are mentioned in Table 2. The method was modified from Zhu et al. (18).

**Table 2:** Sensory evaluation of almond yogurt.

Sensory attribute	Description/Criteria	Score
Appearance	Milky white, glossy, and homogenic	7–9
	Faint yellow, relatively uniform, and glossy	4–6
	Dark and inhomogeneous	1–3
Odor	Obvious pleasant smell that is sustained and no abnormal sour smell	7–9
	Pleasant smell and normal sour smell	4–6
	There is an unpleasant smell	1–3
Taste and acidity	Smooth and soft, sweet and sour balance, not grainy	7–9
	Soft, sour, and slightly grainy	4–6
	Coarse, extremely sour, and unusual taste	1–3
Texture	Uniform and stable, without or with little whey expelled	7–9
	Generally uniform and stable, with a small amount of whey expelled	4–6
	Inhomogeneous and loose, with a large amount of whey expelled	1–3
Overall acceptance	Overall good	7–9
	Acceptable	4–6
	Not recommended to taste	1–3
ITEM	Yogurt Scoring Criteria	Score

#### 2.2.15 Statistical analysis

Each experiment was carried out in triplicate. GraphPad Prism 8 (GraphPad Software, San Diego, CA, United States) was used to analyze the data. Two-way ANOVA was used to determine statistical significance for antioxidant studies, and the findings are represented as mean ± standard deviation. Pearson Correlation test was used for Sensory evaluation studies.

## 3 Results and discussion

### 3.1 Tolerance to simulated gastrointestinal fluid (SGF) of *Enterococcus faecalis* RIT BM_S15-3_ in almond yogurt

Gastrointestinal tolerance of a probiotic is important because it determines its functional efficacy, and only those that can tolerate gastric acidity, bile salts, and digestive enzymes can reach the intestine in live form to deliver health benefits. Various methods have been explored to enhance the survival of probiotics, including co-culture techniques (28) and biopolymer-based complexes for protection (29). Tolerance to SGF also helps identify strain-specific robustness and enables the formulation of probiotic-based food products with advanced stability and functionality (30, 31). Probiotics that tolerate SGF can alleviate digestive discomfort and improve gastrointestinal health, according to the clinical evidence provided by Garvey et al. (32). Hence, tolerance to SGF is a fundamental method for selecting a strain and for its application in the food industry. The current study involves a GI tolerance study of a probiotic strain of *E. faecalis* RIT BM_S15-3_ isolated and characterized from healthy human breast milk (19), inoculated in almond milk, and fermented (Figure 1). This study demonstrated that the strain initially showed a high cell count of 12.13 log CFU/mL in alpha-amylase. The viability declined gradually (from 11.60 (0 min) to 9.88 log CFU/mL (180 min) after exposure to SGF and further declined when it was passed to SIF (9.15 log CFU/mL), but gradually increased to 11.54 (16 h) due to near-acidic pH conditions (6.8–7.0). Overall, the present study showed survival during gastrointestinal conditions and also maintained viable counts above the functional threshold (>10^6^ CFU/mL), thereby supporting potential health benefits *in vivo* (33). For instance, several lactic acid bacteria and Enterococcus spp. have been shown to retain viability within the range of 6–8 log CFU/mL following simulated gastrointestinal transit (34).

**Figure 1:**
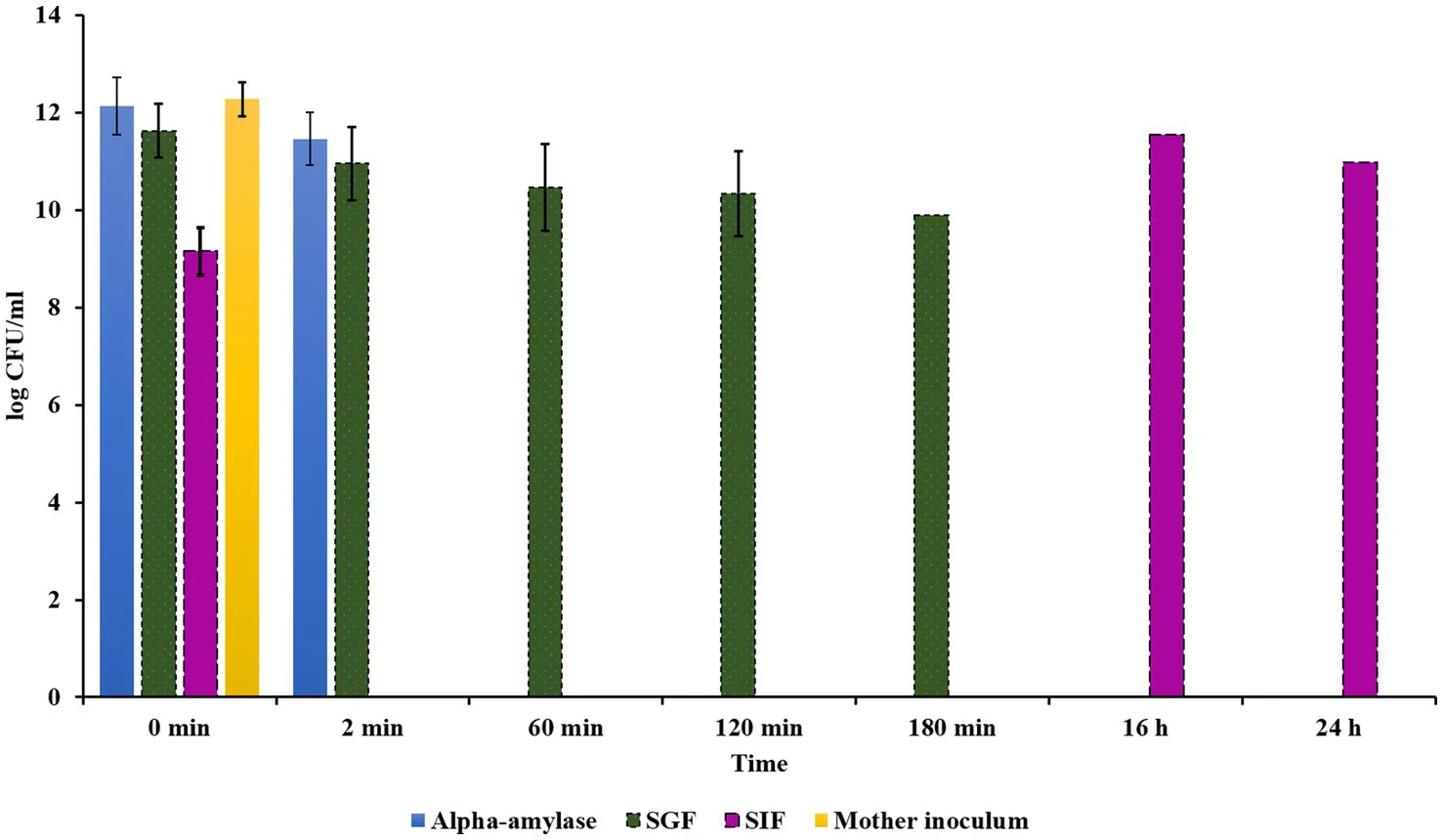
Gastrointestinal tolerance of *E. faecalis* RITBMS_15−3_ in almond yogurt during storage. The error bars represent the mean ± SD of three independent experiments.

### 3.2 Tolerance to simulated intestinal enzymes

The cell viability of *E. faecalis* RIT BM_S15-3_ in simulated intestinal conditions after 48 h of fermentation in almond milk (Figure 2) demonstrated that the strain possessed considerable resistance to gastrointestinal enzymes. Exposure to simulated intestinal fluid containing bile salts and digestive enzymes resulted in a marginal reduction in cell count, yet maintained counts above the minimal therapeutic level generally recommended for probiotic efficacy (≥10^6^–10^7^ CFU/mL) (35). This indicates that almond yogurt is an effective non-dairy probiotic carrier in supporting the intestinal survival of *E. faecalis* RIT BM_S15-3_. The strain showed a viability of 9.56 log CFU/mL at 0 h in almond yogurt, which increased to 10.33 log CFU/mL after 4 h. The pH also changed from 6.80 to 6.32. The higher survival in almond yogurt may be attributed to both the strain’s intrinsic stress tolerance and the protective effect of the almond yogurt matrix. The strain can withstand harsh gastrointestinal conditions, including digestive enzymes and variability in pH. Reports have shown that *E. faecalis* RIT BM_S15-3_ exhibits significant tolerance to bile and simulated intestinal environments, which contributes to its persistence in the gastrointestinal tract (19). Survival in MRS medium showed a small decline compared with almond yogurt. Cell survivability was 8.98 log CFU/mL at 0 h and declined to 8.85 after 4 h. A reduction in pH from 5.75 to 5.42 was observed during this time period. This can be attributed to the lack of a protective matrix in the medium. In both cases, the strain-maintained counts were above the minimal recommended numbers for probiotic efficacy. Furthermore, the relatively low reduction in viable count after enzyme exposure suggests that fermentation may have promoted adaptive stress responses in *E. faecalis* RIT BM_S15-3_. Exposure can lead to physiological adaptations like membrane modification, increased activity of the proton pump, and stress protein production, which further improve tolerance to intestinal conditions. Similar observations have been reported for probiotic enterococci and other lactic acid bacteria in simulated gastrointestinal environments (36). The results from the current study are consistent with reports indicating that plant-based fermented matrices can effectively maintain probiotic viability during gastrointestinal simulation. Compared with dairy substrates, almond yogurt offers additional benefits such as the absence of lactose and cholesterol, making it an attractive option for vegan and lactose-intolerant consumers while still supporting the survival of probiotics (37). Overall, these findings demonstrate fermented almond milk as a protective matrix under simulated intestinal conditions and suggest it may be a promising functional probiotic beverage. However, since some strains of *E. faecalis* are associated with opportunistic pathogenicity, strain-level safety evaluation (38) is essential before commercial or therapeutic application.

**Figure 2:**
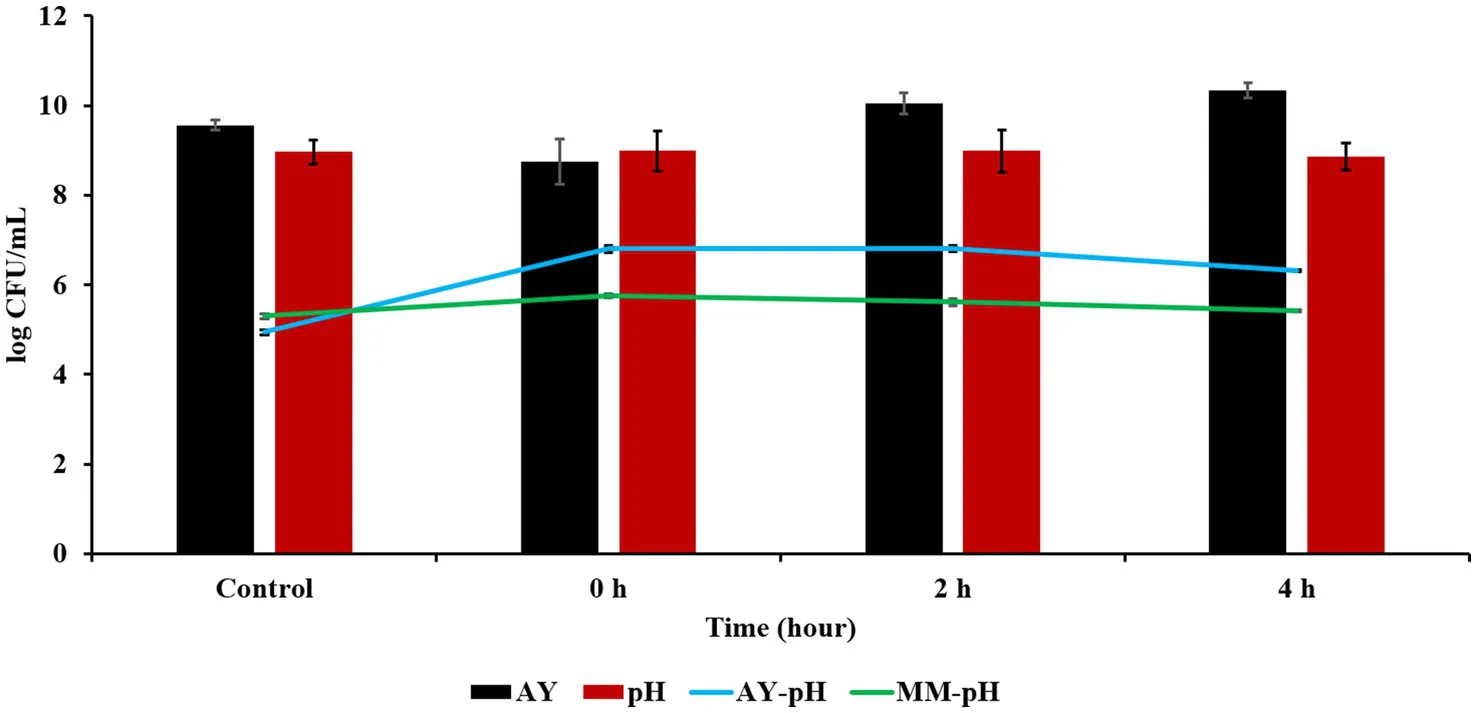
Survival of *E. faecalis* RIT BMS15-3 in almond yogurt (AY) and MRS-MSG medium (MM) under simulated intestinal enzyme conditions at 0, 2, and 4 h of incubation. The lines represent the change in pH of the culture in AY and MM for 4 h. Viable cell counts are expressed as log10 CFU/mL. The data represent the mean ± SD of three different experiments. The control is the cell viability before enzyme treatment.

### 3.3 pH and Total Titratable acidity (TTA)

pH and total titratable acidity (TTA) are key indicators of fermentation progress and storage stability in almond yogurt, as a decrease in pH is usually accompanied by an increase in TTA due to acid production by probiotics. This variability affects sensory quality, texture, and probiotic strain survival (39). Excessive acidification negatively affects probiotic viability and consumer acceptability. Therefore, maintaining a balanced pH and TTA is critical for ensuring extended shelf life. Figure 3 shows the variation of pH and TTA of almond yogurt over 21 days of refrigerated storage. pH gradually declined from 5.85 to 4.28 during this time period, whereas TTA displayed a gradual rise from 0.24 to 1.02% from 0 h to 21 days. The uninoculated milk displayed negligible change (5.85–5.65) in pH and TTA (0.15–0.16) throughout the storage time period. The decline in pH corresponds to active carbohydrate metabolism and acid production during the initial growth phase. Previous reports suggest that during refrigerated storage, TTA increased in symbiotic drinks (40). These results suggest that increased TTA and reduced pH show almond yogurt as an effective non-dairy probiotic beverage.

**Figure 3:**
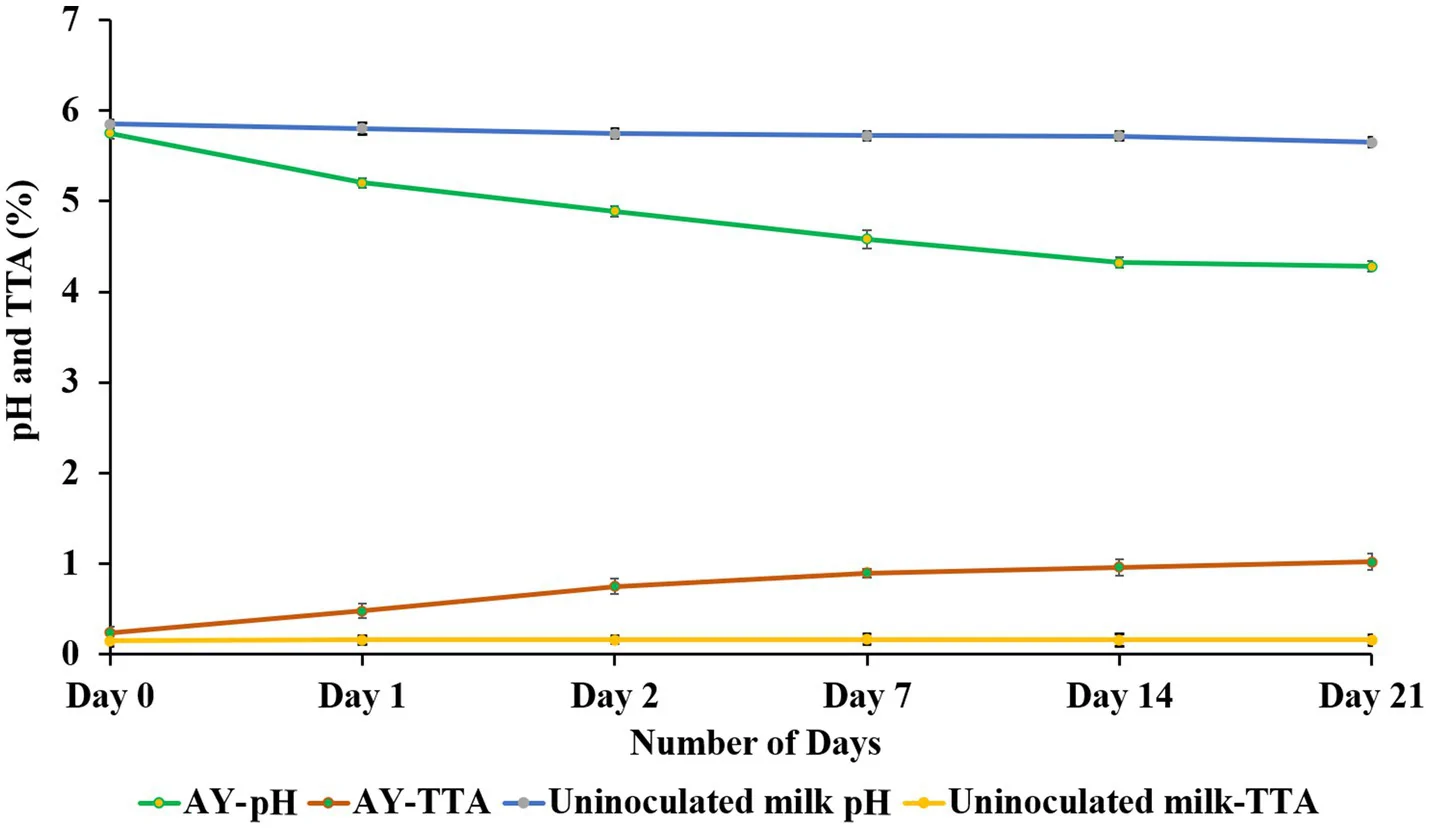
Variation in pH and TTA of AY during refrigerated storage for 21 days. The error bars represent the mean ± SD of three different experiments. The blue and yellow lines represent the pH and TTA of uninoculated almond milk, respectively.

### 3.4 Analysis of antioxidant activity

In beverages, antioxidant activity is an important functional property as it reduces oxidative stress by scavenging free radicals, thereby contributing to better health benefits. The antioxidant activity of almond yogurt was assessed to establish its potential as a value-added functional probiotic food using DPPH and ABTS radical-scavenging assays, and the results showed a concentration-dependent increase in percentage inhibition for both fermented and uninoculated samples. However, the fermented almond yogurt consistently showed higher antioxidant activity than the uninoculated control. Among the concentrations tested, the 50% sample concentration showed 34.80% DPPH scavenging activity, and uninoculated milk showed 32.80% activity, respectively (Figure 4). The DPPH of commercially available dairy yogurt and peanut yogurt were measured for comparison. An activity of 18.58% was shown by dairy yogurt at 50% concentration, whereas 10.76% was exhibited by peanut yogurt at 100% concentration. This revealed the relatively high antioxidant activity of almond yogurt in comparison with commercially available yogurts. Two-way ANOVA revealed significant effects of sample type, concentration, and their interaction (*p* < 0.0001 for all). Bonferroni *post hoc* analysis showed significant differences between almond yogurt and each of the other samples (uninoculated, peanut, and dairy yogurt) at all tested concentrations. ***denotes *p* < 0.001 for the indicated pairwise comparison.

**Figure 4:**
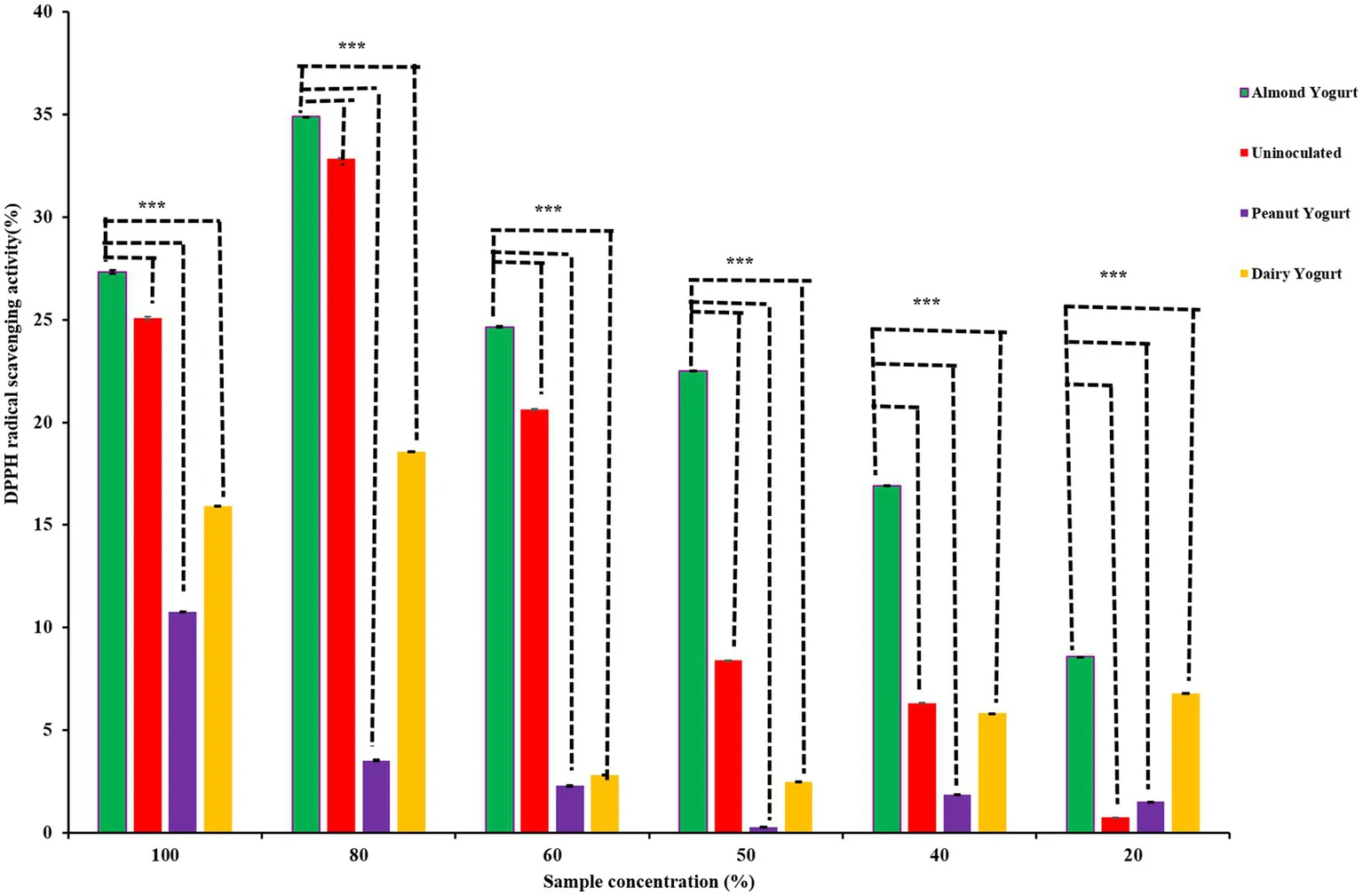
DPPH scavenging activity of fermented almond milk and uninoculated milk in storage conditions, along with peanut and dairy yogurts. Data are presented as mean ± standard deviation (SD) of three independent replicates. Two-way ANOVA revealed significant effects of sample type, concentration, and their interaction (*p* < 0.0001 for all). Bonferroni *post hoc* analysis showed significant differences between almond yogurt and each of the other samples (uninoculated, peanut, and dairy yogurt) at all tested concentrations. *** denotes *p* < 0.001 for the indicated pairwise comparison.

Similarly, the ABTS assay exhibited a consistent increase in total antioxidant activity in almond yogurt compared to uninoculated milk. Different concentrations of almond yogurt were tested to evaluate the antioxidant potential. Among the sample concentrations, the highest free radical scavenging activity was observed at 50% with respect to uninoculated milk, and the lowest activity was found at 40% (Figure 5). Peanut yogurt showed an activity of 15%, whereas dairy yogurt exhibited 6.1% activity at 50% sample concentration. There was a 50% increase in the fermented sample (50% sample concentration) compared with the uninoculated control (25% scavenging activity). Two-way ANOVA revealed significant effects of sample type, concentration, and their interaction (*p* < 0.0001 for all). Bonferroni *post hoc* analysis showed significant differences between almond yogurt and each of the other samples (uninoculated, peanut, and dairy yogurt) at all tested concentrations. ***denotes *p* < 0.001 for the indicated pairwise comparison. This shows the role of fermentation in enhancing the antioxidant activity. In summary, almond yogurt showed a higher antioxidant activity than uninoculated almond milk and the commercially available peanut and dairy yogurts.

**Figure 5:**
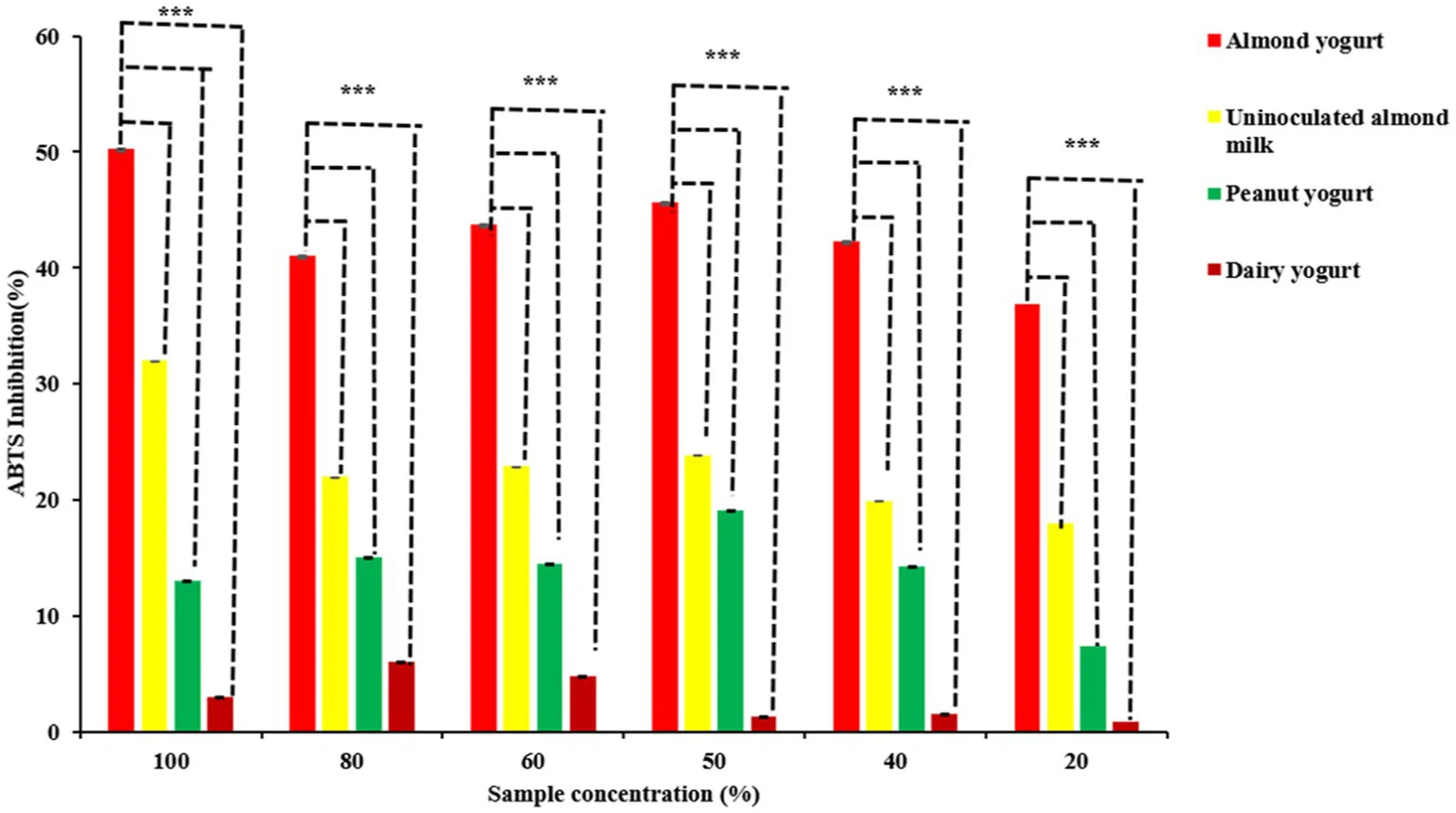
ABTS radical scavenging activity of almond yogurt and uninoculated milk in storage conditions along with dairy and peanut yogurt. Data are presented as mean ± standard deviation (SD) of three independent replicates. Two-way ANOVA revealed significant effects of sample type, concentration, and their interaction (*p* < 0.0001 for all). Bonferroni *post hoc* analysis showed significant differences between almond yogurt and each of the other samples (uninoculated, peanut, and dairy yogurt) at all tested concentrations. *** denotes *p* < 0.001 for the indicated pairwise comparison.

The enhanced antioxidant potential observed in almond yogurt may be attributed to microbial biotransformation of phytochemicals during fermentation. During fermentation, microbial enzymes like proteases, esterases, and *β*-glucosidase hydrolyze complex macromolecules and release bound phenolic compounds, peptides, amino acids, and flavonoids with improved radical-scavenging potential. Almonds are naturally rich in phenolic compounds, flavonoids, and vitamin E, and fermentation improves the availability of these antioxidant constituents. Moreover, metabolites produced during fermentation, including organic acids, exopolysaccharides, and bioactive peptides, may further contribute to the antioxidant potential (41). The higher antioxidant activity of fermented yogurt suggests that fermentation improved the functional quality of the plant-based substrate. Similar results have been reported in fermented plant-based beverages and probiotic yogurts, where fermentation by lactic acid bacteria enhanced DPPH and ABTS radical-scavenging activities, likely due to increased phenolic content and production of bioactive metabolites (42, 43).

### 3.5 Analysis of Total phenolic content (TPC)

TPC is an important indicator of the antioxidant capacity and functional quality of fermented plant-based compounds. Almond milk contains phenolic compounds, including flavonoids and phenolic acids that contribute to antioxidant activity and overall nutritional value (44). Fermentation with probiotic cultures further enhances the bioavailability and release of these compounds through microbial enzymatic activity, increasing the functional efficacy of the final product. Hence, evaluating TPC in almond yogurt during storage provides insight into biochemical transformations occurring during fermentation and the stability of bioactive compounds over time.

The assessment of TPC in almond yogurt during the storage period of 21 days demonstrated an increase in phenolic content compared to uninoculated almond milk used as the control (Figure 6). The fermented samples exhibited relatively higher TPC values (0.048–0.075 mg GAE/g from Day 1 to Day 21) throughout the storage, indicating sustained microbial metabolism and release of phenolic compounds. This increase may be attributed to the enzymatic activities of *E. faecalis* RIT BM_S15-3_, including *β*-glucosidase and esterase enzymes, which hydrolyze complex phenolic fragments and release bound phenolics into more bioavailable forms. Moreover, proteolytic activity during fermentation may contribute to the breakdown of almond protein-polyphenol complexes, thereby increasing phenolic availability (45). The uninoculated almond milk exhibited comparatively lower TPC values throughout storage (0.013–0.048 mg GAE/g from Day 1 to Day 21), suggesting limited biochemical conversion in the absence of fermentation. The enhancement of TPC observed in almond yogurt is consistent with previous studies reporting increased phenolic content in probiotic-fermented dairy products due to microbial biotransformation and improved phytochemical extractability (46, 47). Fermentation-induced acidification may also facilitate the release of phenolic compounds from plant cellular matrices, thereby improving antioxidant potential. Furthermore, the elevated TPC values are consistent with the enhanced antioxidant activity observed in the fermented samples, supporting the role of phenolic compounds as major contributors to free radical scavenging activity. These results indicate that fermentation enhances the phenolic profile and functional properties of almond yogurt, reinforcing its potential as a bioactive and antioxidant-enriched non-dairy functional food.

**Figure 6:**
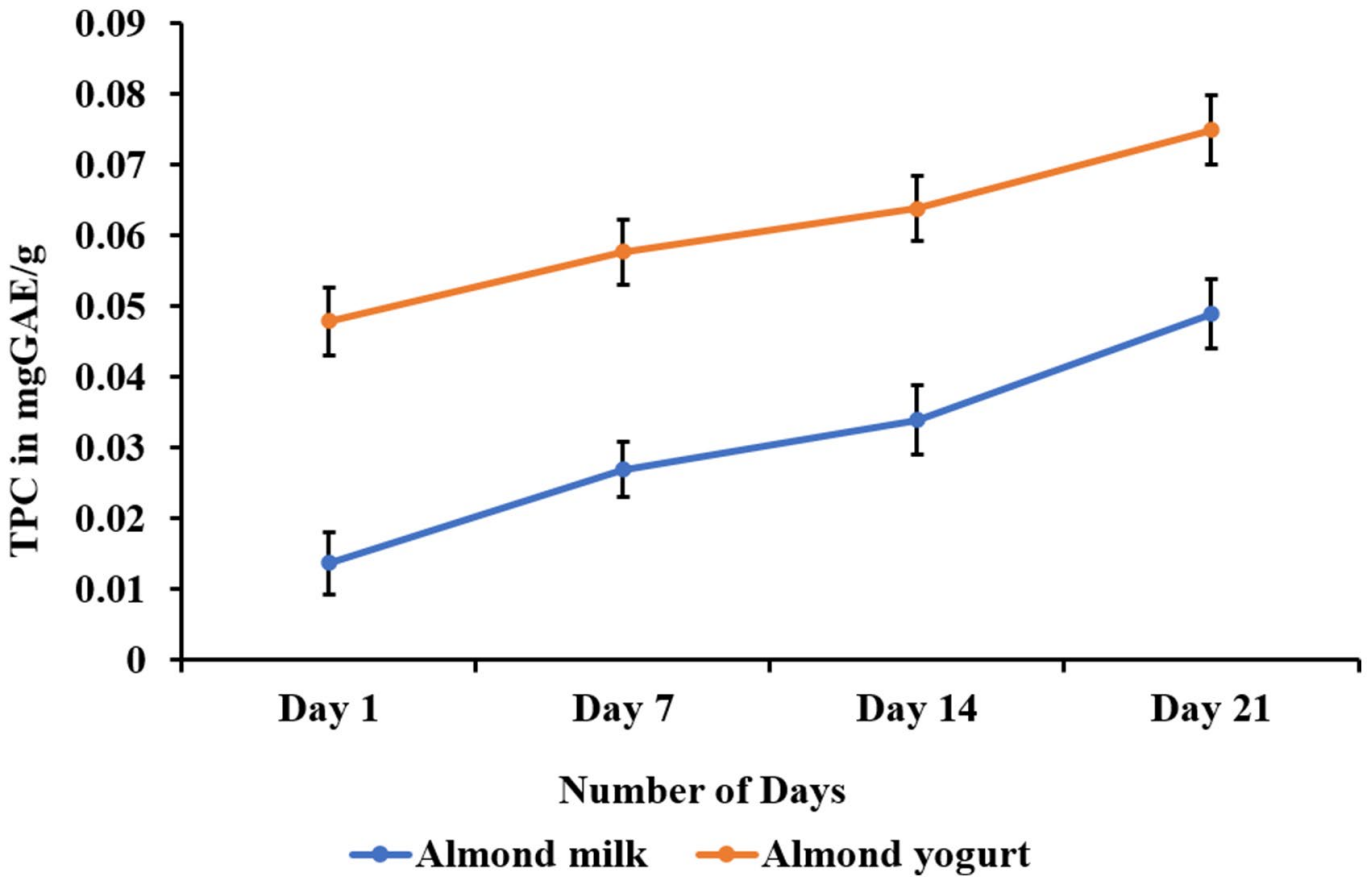
Total phenolic content of almond yogurt inoculated with *E. faecalis* RIT BM_S15-3_ during storage.

### 3.6 Analysis of total flavonoid content (TFC)

TFC is an important factor in the antioxidant potential and functional quality in fermented plant-based products. Almond milk naturally consists of flavonoids and other polyphenolic compounds that are responsible for antioxidant activity, oxidative stability, and associated health-promoting effects. Fermenting with the *E. faecalis* RIT BM_S15-3_ culture can affect the availability of flavonoids through microbial enzymatic activity, resulting in the release of bound flavonoids and conversion into more bioactive forms (47). Hence, monitoring TFC during the storage period provides insight into biochemical transformations, functional enhancement, and the stability of fermented almond yogurt over its shelf life.

Determining TFC in almond yogurt showed a progressive increase (1.17 to 1.85 mg QE/g from Day 1 to Day 21) throughout the storage period, with higher flavonoid levels observed in fermented samples compared to uninoculated almond milk used as the control (Figure 7). The increase in flavonoid content during storage suggests microbial metabolism and sustained enzymatic biotransformation of flavonoid-associated compounds. The uninoculated milk showed comparatively lower and stable TFC values (0.12 to 1.12 mg QE/g from Day 1 to Day 21) when compared to the yogurt. This indicates limited flavonoid release in the absence of fermentation. The enhanced flavonoid content in almond yogurt may be attributed to the activity of microbial enzymes that hydrolyze complex flavonoid conjugates and release bound flavonoids into bioavailable forms. Moreover, fermentation-induced acidification may facilitate the breakdown of plant cell wall structures and interaction between proteins and polyphenols, thereby improving flavonoid extractability and stability. These results are consistent with previous reports showing that probiotic fermentation enhances flavonoid release and antioxidant potential in plant-based yogurts through microbial bioconversion and enhanced accessibility to phytochemicals (48). Increased flavonoid content during fermentation is associated with improved radical scavenging activity and overall antioxidant functionality (49). Overall, the findings indicate that probiotic fermentation improves the bioavailability of flavonoids and the functional quality in almond yogurt, proving its potential as an antioxidant-rich and bioactive non-dairy functional food.

**Figure 7:**
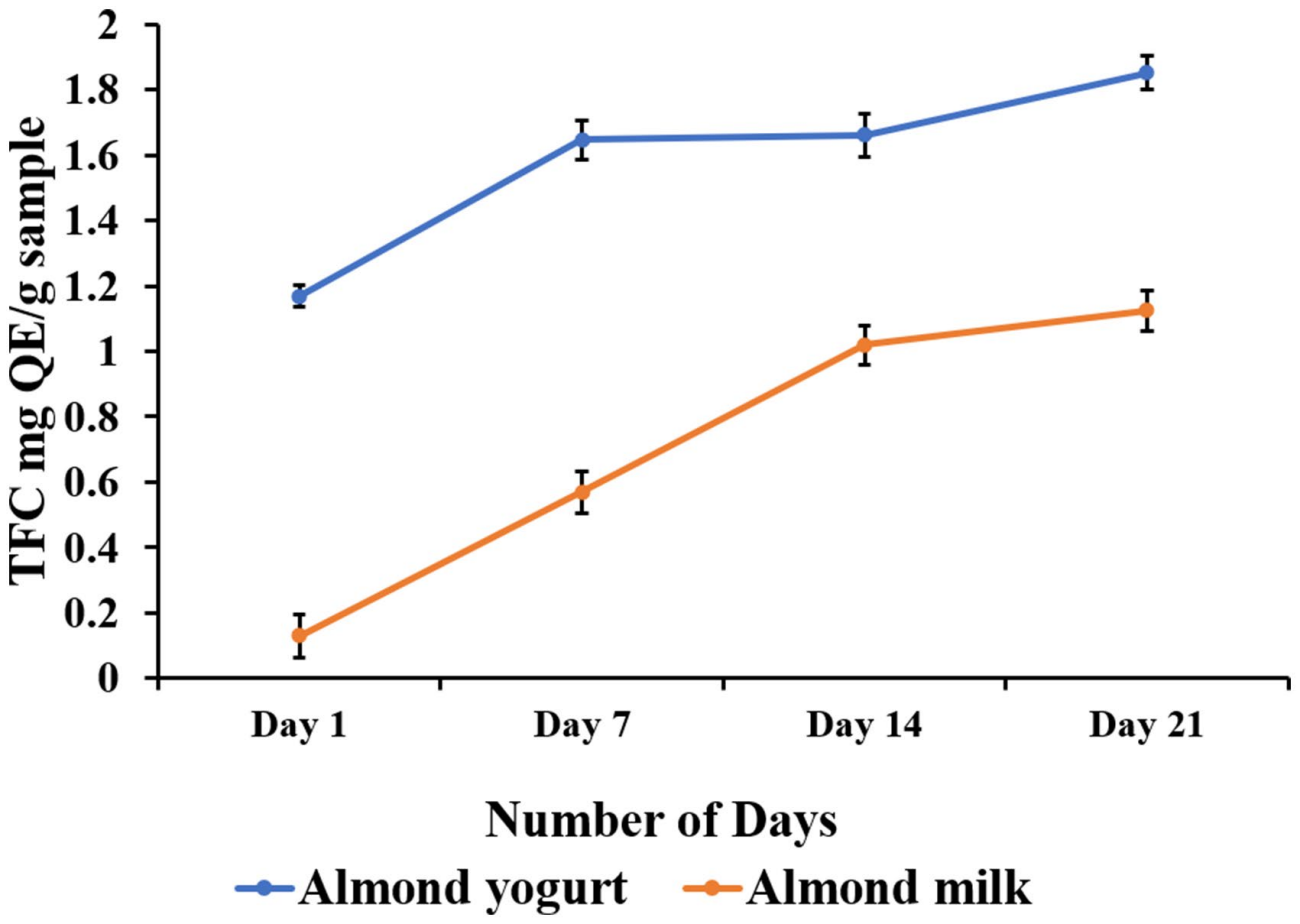
Total flavonoid content of almond yogurt inoculated with *E. faecalis* RIT BM_S15-3_ during storage.

### 3.7 Multiple factor evaluation of GABA production in almond yogurt

#### 3.7.1 Effect of fermentation time on GABA production and microbial growth dynamics

Fermentation time is an important parameter in almond yogurt production as it determines the stages of microbial growth and GABA production. It also plays a major role in the yogurt texture because it is affected by the acidification at the fermentation endpoint. The present study depicts GABA production, along with microbial growth dynamics and acidification with respect to fermentation time (Figure 8). The data indicate that GABA accumulation showed an increasing trend from the early to late fermentation stage. After 24 h, production increased continuously and reached a maximum of 29.84 mM GABA at 48 h. Microbial growth (Figure 8B) followed a rapid increase in cell count till 24 h (11.50 log CFU/mL), after which there was a decline in growth, as cells began to decay after 32 h (11.44 log CFU/mL), and at 48 h, it decreased to 11.28 log CFU/mL.

**Figure 8:**
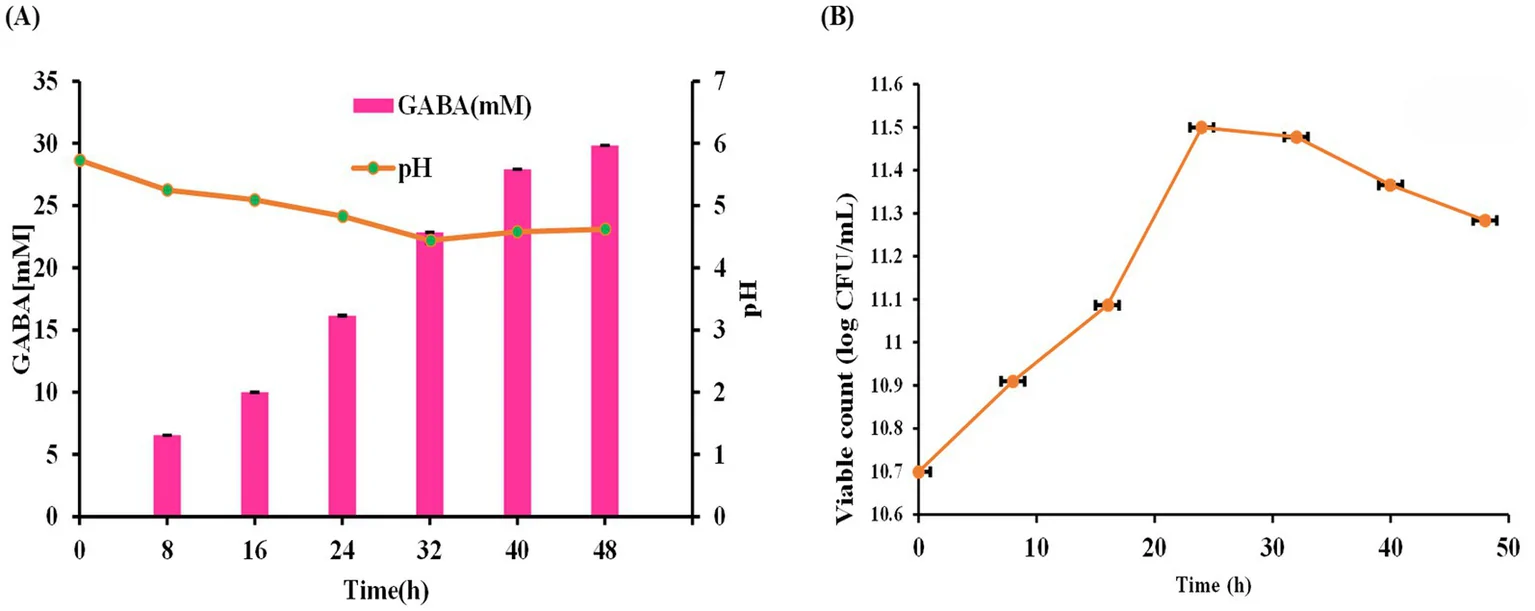
**(A)** Effect of fermentation time on GABA yield and acidity. **(B)** Effect of fermentation time on microbial growth. The error bars represent the mean ± SD of three independent experiments.

The temporal profiles of GABA accumulation and viable cell count revealed that maximum GABA production did not coincide with maximum biomass formation. While the viable cell count reached its maximum of 11.50 log CFU/mL at 24 h and subsequently declined, GABA accumulation continued and reached its maximum concentration of 29.84 mM at 48 h (Figure 7). This suggests that GABA biosynthesis was not directly proportional to cell proliferation and that GAD-mediated GABA production continued during the later fermentation stage despite the decline in viable cell count. The acidification was balanced throughout the fermentation process, and the pH at 48 h of fermentation was 4.62. Therefore, fermentation time affected GABA yield. In the early fermentation period, the components in the yogurt were intensively used to increase biomass, and GABA yield was limited. It began to increase continuously after 24 h of fermentation (Figure 8A), as the bacteria entered the stationary phase and started producing GABA. Yogurt pH decreased until 24 h (4.83), and the final pH of 4.62 was higher than that of commercial yogurts, which range from 4.1 to 4.3. The simultaneous changes in pH and GABA accumulation further indicate a dynamic relationship between acidification and GABA biosynthesis. The pH decreased substantially during the period of active microbial growth, from approximately 5.8 at the beginning of fermentation to 4.83 at 24 h, whereas only a moderate further decrease to 4.62 was observed by 48 h. Conversely, GABA concentration continued to increase markedly during this time period. This pattern may reflect the contribution of the GAD system to acid-stress adaptation, where glutamate decarboxylation consumes a proton and generates GABA, thereby helping maintain intracellular pH homeostasis. This result is due to GABA production, which consumes hydrogen ions and helps neutralize the pH decline resulting from bacterial growth (18). This could prevent over-acidification of the yogurt and improve shelf life. Regarding the viable cell count, *E. faecalis* RIT BM_S15-3_ showed good growth characteristics. Even in the early fermentation stage (8 h), the viable cell number reached 10.90 log CFU/mL (Figure 8B), which is more than that of commercial yogurt. After 24 h (11.50 log CFU/mL), the number of microorganisms began to decline to 11.36 log CFU/mL due to a progressive decrease in nutrient levels in the yogurt, which could no longer support a high level of cell growth. Meanwhile, cell growth and the reduction in pH mutually promote each other. That is, the bacteria convert sugars present in the almond to acid, decreasing the pH and creating a relatively acidic environment that supports growth. This induced glutamic acid decarboxylase (GAD) activity to catalyze the conversion of L-MSG to GABA, confirming the GABA accumulation trend (18). Thus, 48 h of fermentation was recommended to achieve a sufficient GABA yield and an appropriate pH during yogurt formation. Thus, the combined profiles of viable cell count, pH, and GABA accumulation indicate a dynamic fermentation balance in which early-stage microbial proliferation and acidification are followed by continued GAD-mediated GABA accumulation during the later fermentation phase.

#### 3.7.2 Effect of fermentation temperature on GABA production, microbial growth, and pH

Fermentation temperature affects microbial growth and exopolysaccharide (EPS) formation, influencing bacterial acidity, which may further affect the yogurt texture and GABA yield. The GABA production, pH of yogurt, and the growth of *E. faecalis* RIT BM_S15-3_ are shown in Figure 9. Maximum GABA yield of 29.60 mM was found to be at 37 °C (Figure 9A). Microbial growth exhibited a non-monotonic temperature-dependant pattern, with relatively low growth at 25 °C (11.80 log CFU/mL), increasing to a maximum at approximately 35 °C (12.6 log CFU/mL), followed by a decline at higher temperatures. The pH at different temperatures ranged from 4.50 to 5.01. The results suggested that the fermentation temperature influenced GABA yield and growth. GABA production increased at 37 °C; after that, as temperature increased, production declined (Figure 9A). The increase in temperature aided bacterial growth and proliferation. Also, the optimum catalytic temperature required for glutamic acid decarboxylase (GAD), which converts L-MSG into GABA, is 37 °C. At temperatures exceeding 37 °C, the bacterial cells experienced thermal stress and diminished metabolic activity. Further, excessive heating altered the almond protein network through increased protein aggregation and weakened gel structure integrity, leading to reduced water retention and lower WHC (50, 51). Furthermore, higher temperatures may alter the microstructure of the almond protein matrix, resulting in a less homogeneous distribution of microbial cells and potentially increasing the resistance to mass transfer for GABA production. This also explains the microbial growth trend (Figure 9B). The final pH of the fermented yogurt is determined by the balance between GABA biosynthesis, which consumes intracellular H^+^ ions using glutamate decarboxylation, and the metabolic activity of probiotic bacteria, which generates organic acids and leads to acidification. Consequently, the effect of fermentation temperature on the final pH of almond yogurt was not pronounced (Figure 9A).

**Figure 9:**
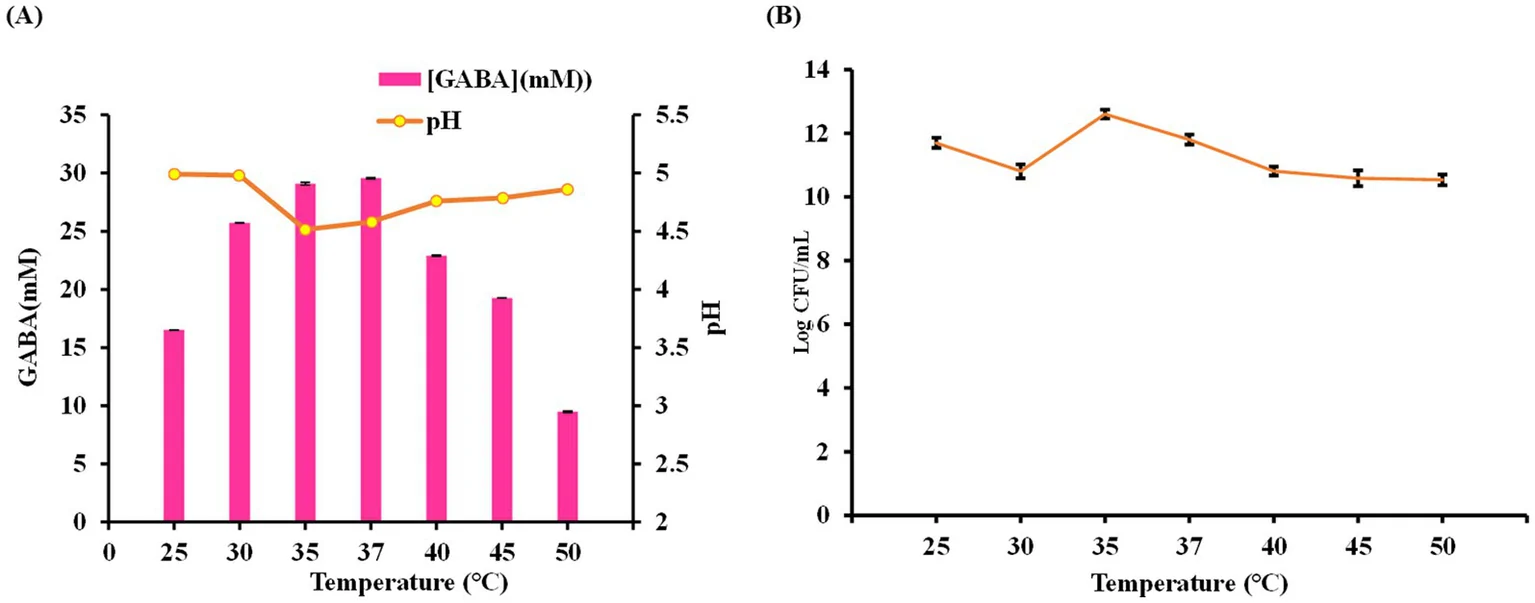
**(A)** Effect of fermentation temperature on GABA yield and acidity. **(B)** Effect of fermentation temperature on microbial growth. The error bars represent the mean ± SD of three independent experiments.

Based on GABA production, microbial growth, and physicochemical characteristics, 37 °C was the optimum fermentation temperature for GABA-enriched almond yogurt. This temperature is comparable to commercial yogurt fermentation processes, suggesting that the production of GABA-enriched almond yogurt could be readily integrated into pre-existing fermentation systems without further modifications to processing conditions or additional energy requirements.

#### 3.7.3 Effects of L-MSG on GABA production, microbial growth, and pH

The concentration of L-MSG, the direct precursor for GABA biosynthesis, influenced GABA production in fermented almond yogurt. GABA yield increased progressively with increasing concentration of L-MSG up to 3 g/L, suggesting enhanced substrate availability for GAD-mediated conversion of glutamate to GABA. At this concentration (3 g/L), the substrate was readily utilized by the *E. faecalis* RIT BM_S15-3_ culture, resulting in efficient bioconversion and a corresponding increase in GABA yield. Sufficient precursor availability, such as carbon flux through the GABA shunt pathway, thereby promotes GABA synthesis. The highest GABA concentration was observed at 3 g/L L-MSG supplementation, indicating that this level provided an optimal balance between substrate availability and enzymatic catalytic capacity. Similar reports have been documented in lactic acid bacteria where moderate glutamate supplementation enhances GABA biosynthesis (18). Beyond 3 g/L, a reduction in GABA yield was observed. This is due to substrate saturation of the GAD enzyme system, where the catalytic sites become fully occupied and additional substrate cannot be effectively converted. Moreover, excessive glutamate concentrations may inhibit cell metabolism or induce feedback regulation mechanisms that limit GAD activity and GABA accumulation. The almond milk matrix also contributes to this response by influencing nutrient availability and bacterial metabolic activity.

L-MSG, a substrate of GAD, is converted into GABA by *E. faecalis* RIT BM_S15-3._ Even though some probiotics can synthesize GABA from a precursor substance within the cell, the amount of GABA produced is very limited. Supplementing the fermentation liquid with L-MSG is required to achieve sufficient GABA yield. GABA production, pH, and microbial growth with respect to changes in L-MSG concentration in g/L are shown in Figure 10B. The data showed an increasing trend in GABA production up to 3 g/L, followed by a gradual decrease at 3.5 and 4.0 g/L. Thus, the L-MSG concentration affected GABA yield. GABA production exhibited a rapid increase when the addition of L-MSG was less than 3 g/L (Figure 10A). Almond milk consists of proteins, carbohydrates, minerals, and phenolic compounds that aid probiotic growth, but excess MSG supplementation could alter the osmotic environment and affect cell physiology. Thus, the decline in GABA production at higher L-MSG concentrations indicates that precursor availability alone does not determine GABA production, and optimal enzyme-substrate interactions are sufficient to maximize GABA biosynthesis. Based on the results of the current study, 3 g/L L-MSG was identified as the optimum concentration for GABA-enriched almond yogurt production.

**Figure 10:**
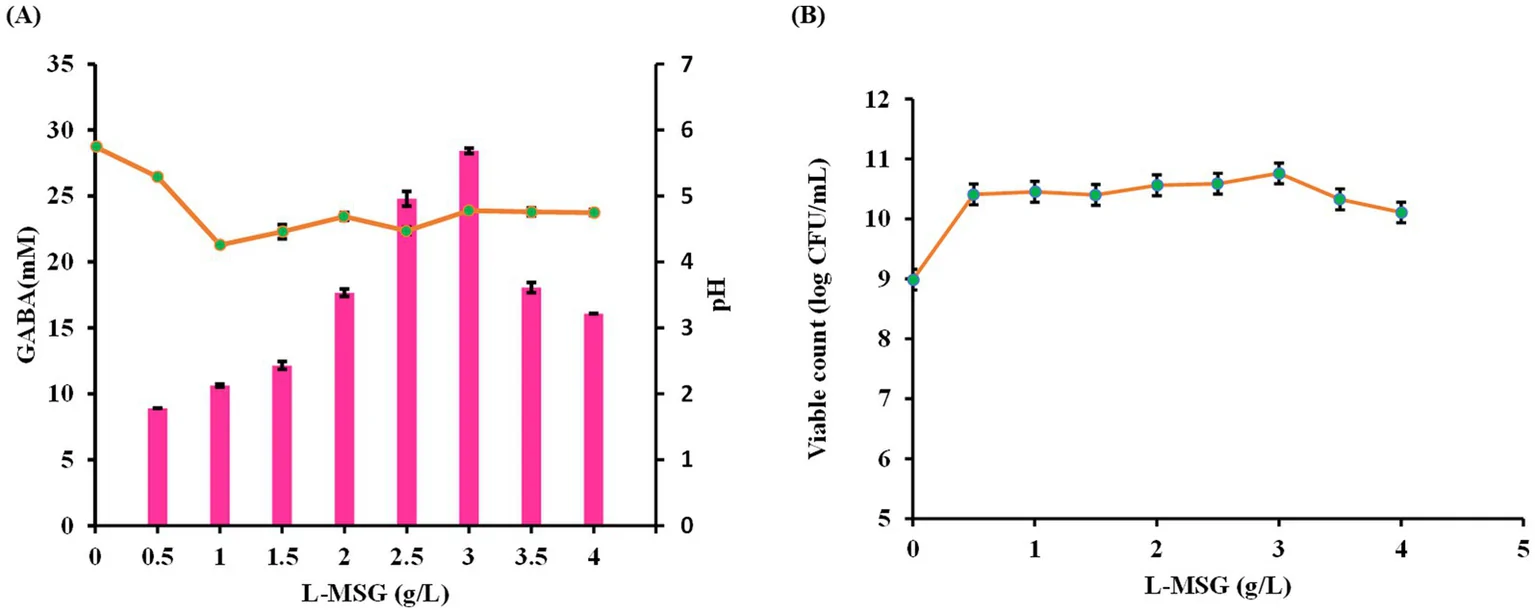
**(A)** Effect of L-MSG on GABA yield and acidity. **(B)** Effect of L-MSG on microbial growth. The error bars represent the mean ± SD of three independent experiments.

The OFAT experiments identified 48 h, 37 °C, and 3 g/L L-MSG as the levels associated with the highest production for fermentation time, temperature, and L-MSG concentration, respectively. These selected levels were subsequently combined in a confirmatory experiment. Under the combined conditions of 48 h fermentation at 37 °C with 3 g/L L-MSG and 10% (v/v) inoculum, a maximum GABA concentration of 29 mM was obtained.

### 3.8 Evaluation of almond yogurt

#### 3.8.1 WHC

WHC represents an emulsion gel’s ability to retain the serum required for the quality of fermented yogurt, as it influences consumer acceptability, and this indicates the stability of the system (52). The WHC was measured throughout the fermentation and storage periods for the GABA-containing almond yogurt to estimate phase separation. Figure 11 shows the relationship between WHC and fermentation. Fermentation time influenced WHC throughout the fermentation period. However, during storage, WHC did not change significantly over time. The WHC of almond yogurt showed a progressive increase during fermentation and remained relatively stable throughout the refrigerated storage. This increase can be attributed to acid-induced almond protein aggregation and the development of a protein-lipid network. The decrease in pH during fermentation promoted intermolecular interactions among the almond proteins, resulting in a denser gel structure with better water-binding ability. Further, thermal processing before fermentation may induce partial protein denaturation, exposing hydrophilic groups that can retain additional water within the gel matrix. Similar observations were reported in almond-based fermented products, where increased protein aggregation and gel network formation resulted in enhanced WHC and reduced serum separation (52). Minimal serum separation was observed during refrigerated storage, indicating the stability of the fermented almond protein network. The maintained WHC can be attributed to structural rearrangement and strengthening of the protein matrix, which improved water immobilization within the gel structure. Previous studies on almond yogurt have reported decreased serum separation and enhanced water retention during storage due to the formation of protein aggregates and stable gel networks (53). The WHC was comparable to that of dairy yogurt studied by Zhu et al. (18).

**Figure 11:**
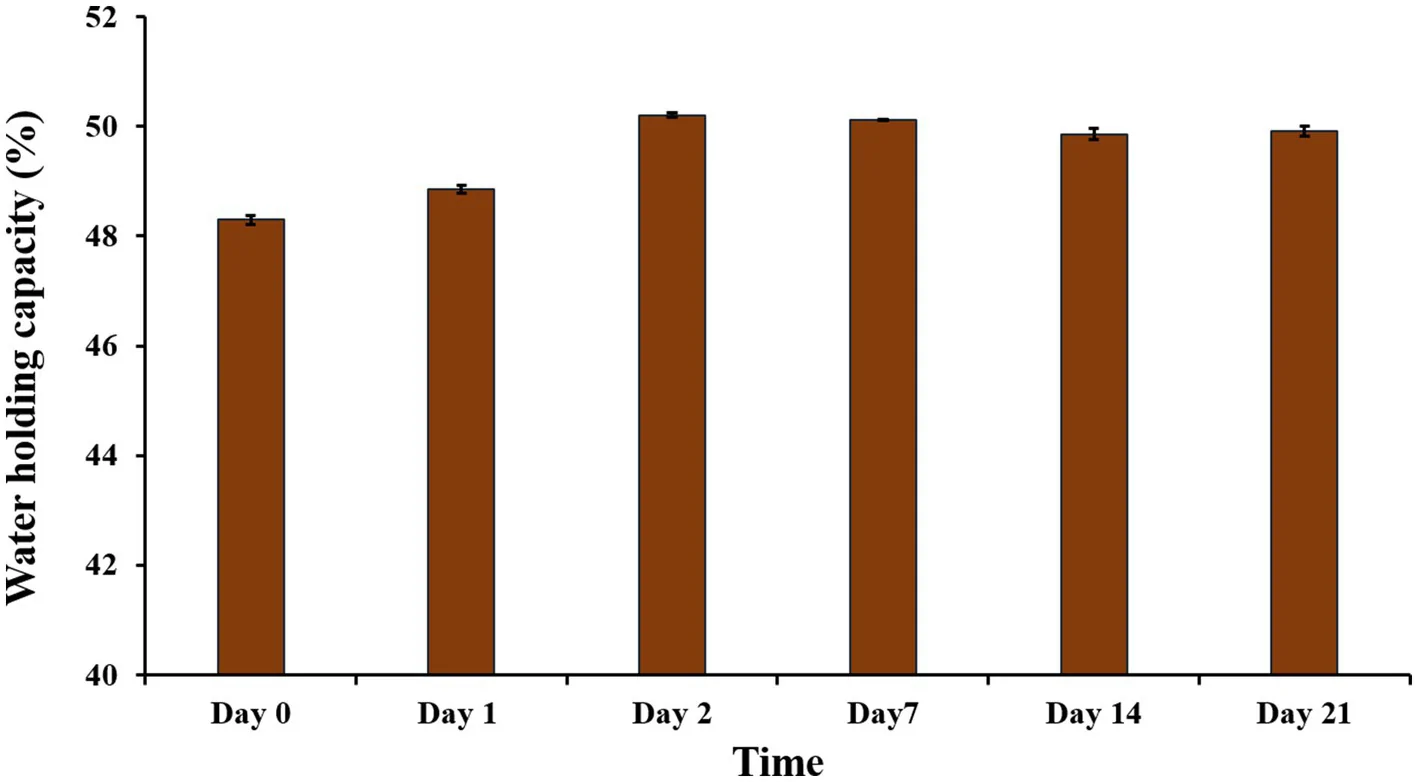
The WHC during fermentation and storage time. The fermentation period was from 0 h to day 2, and the storage period was from day 2 to day 21. The error bars represent mean ± *SD* of three independent experiments.

#### 3.8.2 Viscosity

The viscosity of the almond yogurt increased progressively during fermentation. It remained stable throughout refrigerated storage, reaching a maximum value of 2,327 cP (Figure 12). The increase in viscosity indicates the development of a structured gel matrix during fermentation and reflects improved textural properties of the product. Similar trends have been reported in plant-based fermented products, where acidification enhances protein aggregation and gel formation, increasing viscosity and consistency. The increased viscosity observed in almond yogurt can be attributed to acid-induced aggregation of almond proteins and the formation of a three-dimensional protein network. As fermentation progressed, production of organic acids by the bacteria reduced electrostatic repulsion between protein molecules and facilitated protein–protein interactions. These interactions improved the formation of a denser gel structure capable of entrapping water and suspended particles, thereby increasing resistance to flow and increasing viscosity. Furthermore, microbial exopolysaccharides (EPS), produced by *E. faecalis* RIT BM_S15-3_, may have contributed to enhanced viscosity by increasing water retention and strengthening the gel matrix. EPS improve rheological properties of fermented foods through their water-binding capacity and interactions with proteins, resulting in improved texture. The stability of viscosity during refrigerated storage indicates that the almond yogurt maintained its structural integrity and showed minimal breakdown of the gel network over time (54). This result is consistent with the stable WHC and decreased serum separation commonly associated with plant-based yogurt gels. The viscosity values obtained in the current study indicate the successful development of a fermented almond yogurt with a good texture and storage stability.

**Figure 12:**
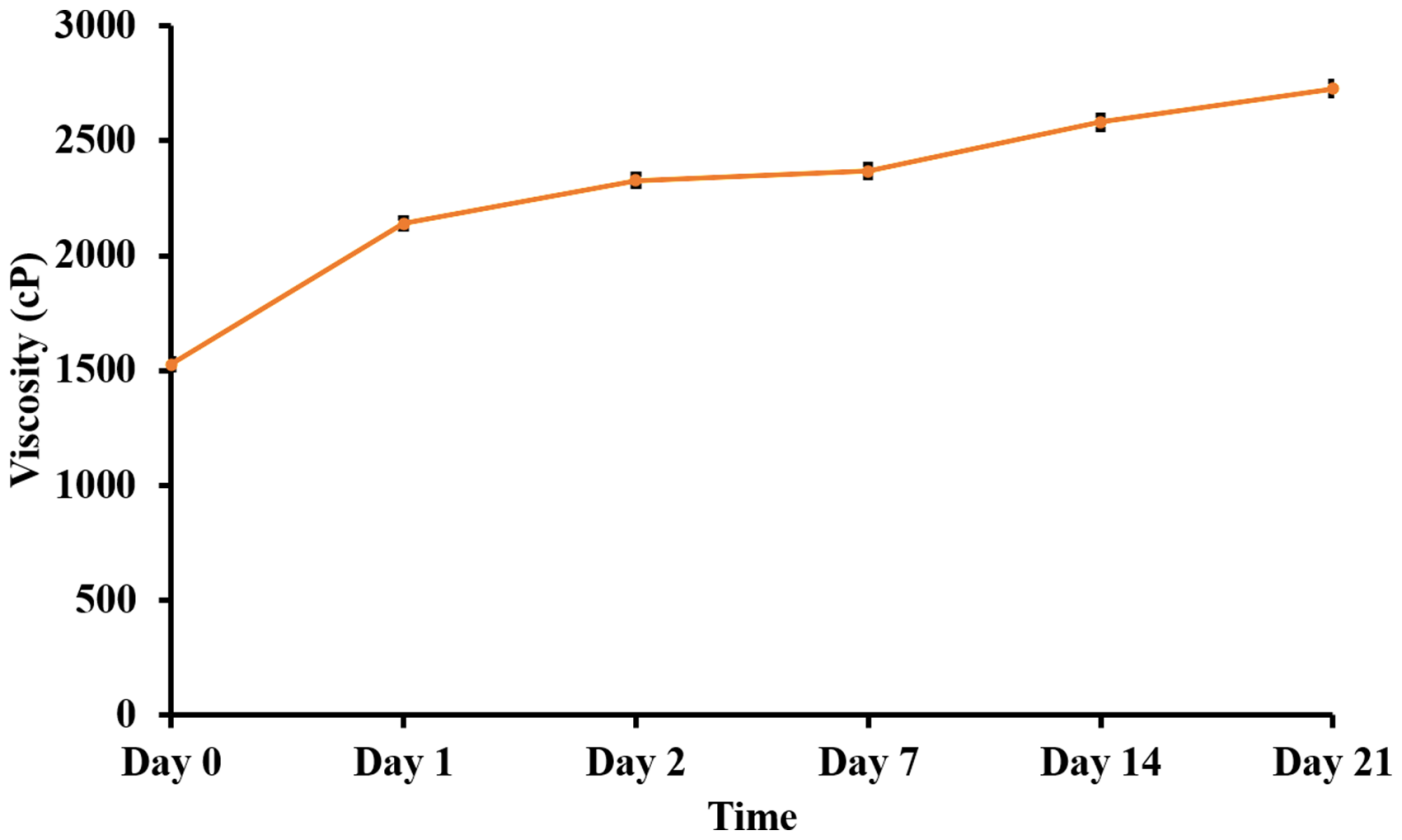
Viscosity of GABA-enriched yogurt during the storage stability test.

#### 3.8.3 WGS-based safety assessment

The safety assessment of *E. faecalis* RIT BM_S15-3_ was considered at the strain level. Although *E. faecalis* is frequently associated with food and commensal environments, the species also includes opportunistic clinical isolates possessing variable combinations of virulence and antimicrobial resistance determinants. Therefore, species identification alone is not sufficient to establish the safety of a strain intended for potential food application. In the present study, we used whole-genome sequencing (WGS) to complement the phenotypic safety characteristics previously established for RIT BM_S15-3_ (19).

WGS analysis revealed the absence of the acquired glycopeptide resistance genes *vanA* and *vanB.* Several clinically relevant virulence-associated determinants, including *esp., cyl, efaA, hyl, ace, ebpA, ebpB, ebpC, fsrA, fsrB, fsrC, sprE*, and genes belonging to the cytolysin operon, were not detected in the genome. The absence of these determinants provides evidence that *E. faecalis* RIT BM_S15-3_ does not possess several of the major virulence-associated genetic features commonly investigated during the safety assessment of *E. faecalis* strains.

However, the genomic analysis also detected *gelE* and *asa1*. The identification of these genes was therefore considered in conjunction with the phenotypic characteristics of the strain rather than interpreted as direct evidence of pathogenicity. Specifically, although *gelE*, which is associated with gelatinase production, was detected in the genome, *E. faecalis* RIT BM_S15-3_ was shown to be phenotypically non-hemolytic and gelatinase-negative (supplementary Figure S1), with no zone of hydrolysis observed on MRS agar containing 3% gelatin following incubation at 37 °C for 24 h. Notably, the *fsrA/fsrB/fsrC* quorum-sensing regulatory operon, which is involved in the regulation of *gelE* expression, was also absent from the genome. The absence of this regulatory system may contribute to the lack of detectable gelatinase activity despite the presence of *gelE* under the conditions tested. This observation indicates that the genomic presence of *gelE* did not correspond to detectable gelatinase activity. Such genotype–phenotype discordance emphasizes the importance of integrating genomic and phenotypic evidence while evaluating virulence-associated characteristics at the strain level. The presence of *asa1*, which encodes an aggregation substance-associated determinant, was likewise considered as a potential safety-related feature requiring further evaluation rather than as an independent indication of pathogenicity.

The previously established phenotypic safety assessment further demonstrated that *E. faecalis* RIT BM_S15-3_ was non-hemolytic and gelatinase-negative (19). These findings provide favorable phenotypic characteristics; however, they do not exclude the presence of other virulence-associated traits. The present WGS analysis therefore provides an additional level of strain-specific characterization and identifies both the absence and presence of relevant genomic determinants.

Antimicrobial susceptibility testing previously performed for *E. faecalis* RIT BM_S15-3_ demonstrated susceptibility to vancomycin, amikacin, linezolid, tetracycline, rifamycin and ampicillin, while resistance was observed to azithromycin, ciprofloxacin, erythromycin and penicillin-G (19). This result has been provided as Supplementary Table S1 to complement the WGS-based assessment. Notably, the phenotypic susceptibility to vancomycin is consistent with the absence of the acquired *vanA* and *vanB* genes in the present genome. However, the detection of the aminoglycoside resistance-associated determinant *aac(6′)-aph(2″)* indicates that the strain cannot be considered completely devoid of antimicrobial resistance determinants. Collectively, *E. faecalis* RIT BM_S15-3_ exhibited a strain-specific safety profile characterized by the absence of *vanA* and *vanB* and various major virulence-associated determinants, together with previously demonstrated non-hemolytic and gelatinase-negative phenotypes. Thus, the combined genomic and phenotypic evidence supports its further evaluation as a potential food-associated GABA-producing strain, while recognizing that additional strain-specific, regulatory, and *in vivo* safety assessments would be required before application in food products.

Overall, the genome analysis demonstrated that *E. faecalis* RIT BM_S15-3_ lacked numerous virulence-associated determinants commonly reported in clinical *E. faecalis* isolates. The study by Pieniz et al. (55) also did not indicate the presence of *vanA* and *vanB* genes in the LAB species *E. durans* LAB18, which was declared safe for food applications. Conversely, Moghimi et al. (56) isolated Lactobacilli from traditional Iranian yogurt, and seven tested positive for the *vanA* gene among the 24 isolates. These results provide an initial overview of the genome and the safety-related characteristics of the isolates and warrant further interpretation in conjunction with phenotypic observations.

#### 3.8.4 Sensory evaluation of the GABA-enriched yogurt

Sensory assessments can provide an overall description of a yogurt. This study analyzed appearance, odor, taste, acidity, texture, and overall acceptance (Figure 13). The results showed that overall acceptance was not significantly different between GABA-enriched yogurt and commercial yogurt. The appearance, odor, taste, acidity, and texture of the almond yogurt were more acceptable than those of the peanut yogurt and slightly less accepted than dairy yogurt. Overall acceptance of commercial dairy yogurt was slightly better than that of almond. The selected commercial yogurts (peanut and dairy) were common and popular in the Indian market. The selected commercial yogurts (peanut and dairy) were common and popular in the Indian market. These results suggested that overall, GABA-enriched almond yogurt (7.52) was competitive in sensory attributes. It is further expected that the health benefits conferred by GABA would improve commercial dairy yogurt (8.36) and be superior to peanut yogurt (7.32) on the market in terms of its ranking.

**Figure 13:**
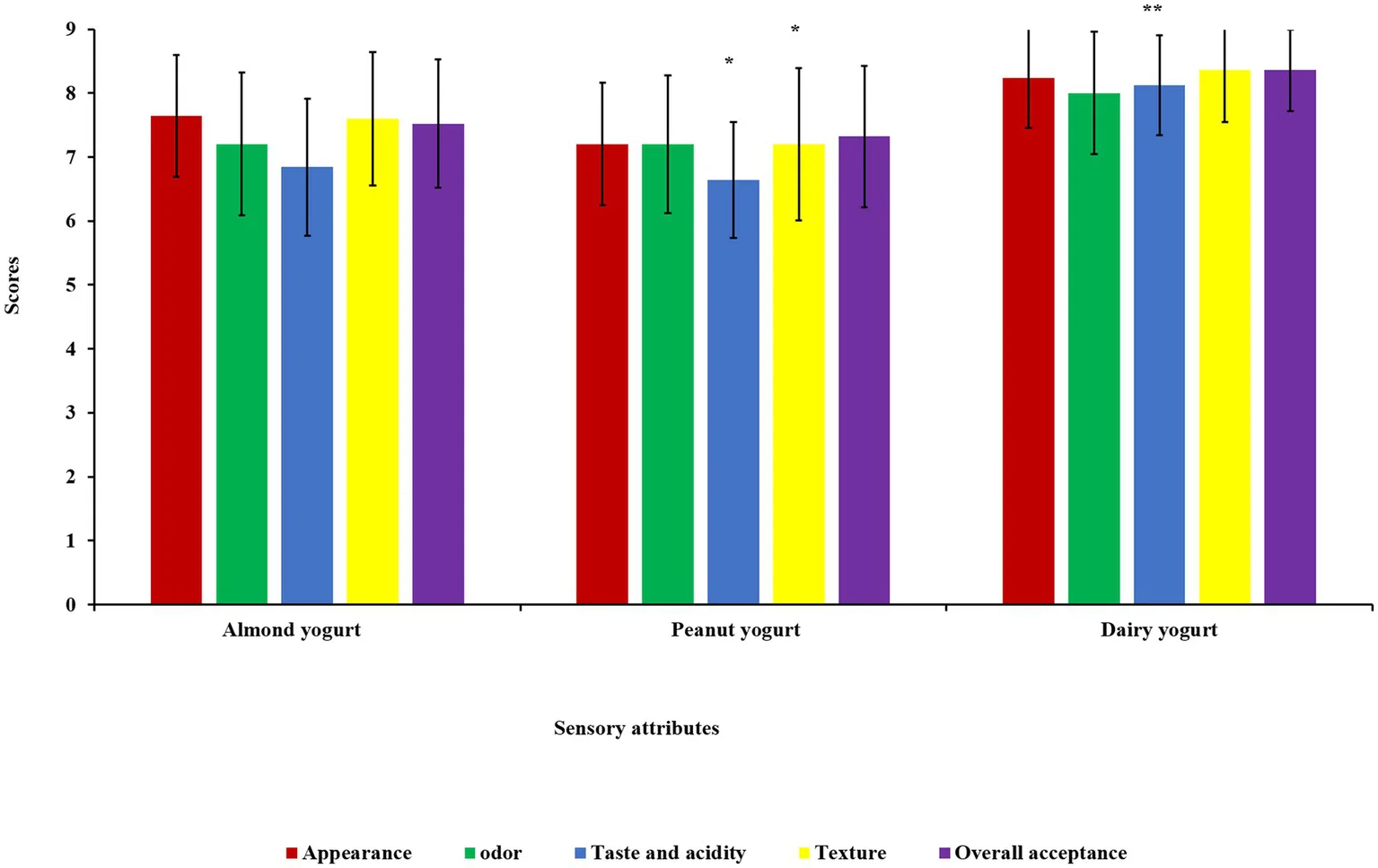
Sensory evaluation scores of almond yogurt, peanut yogurt, and dairy yogurt for appearance, odor, taste and acidity, texture, and overall acceptance. Data are represented as mean ± SD. Pearson correlation analysis was performed to assess the sensory attributes. Significant correlations were observed for taste and acidity of peanut yogurt (*p < 0.05) and taste and acidity of dairy yogurt (**p < 0.01).

#### 3.8.5 Determination of viable bacterial count in GABA-enriched yogurt during storage

The viability of probiotics in yogurt is critical throughout the storage period, as it is required to ensure it delivers the health benefits. The efficacy of probiotics depends on maintaining sufficient cell viability until consumption (57). Yogurt acts as a supportive matrix due to its antioxidant content, which can enhance the survival of probiotics (58). A study on GABA-enriched yogurt observed that total fermentation time is essential to yogurt production, as it determines the microbial growth stage and GABA production (18). However, prolonged storage or processing can reduce cell viability, highlighting the need for protective strategies such as micro- or nanoencapsulation (59). Hence, monitoring probiotic survival in almond yogurt is vital for consumer acceptance, shelf life, and proper functionality of the product. In the current study, the viable cell count of *E. faecalis* RIT BM_S15-3_ was evaluated for 21 days after fermentation. Figure 14B shows the viability from 0 to 21 days with respect to the mother inoculum. Day 1 represents the fermentation phase, and Days 2 to 21 indicate the storage time period. During the fermentation period, the strain showed high viability (11.60 log CFU/mL), confirming active growth and adaptation to the almond matrix, whereas uninoculated milk showed no growth, confirming sterility. This aligns with the known fermentative potential of Lactobacillus species, which use lactose and other carbohydrates to produce lactic acid, reducing pH and stabilizing the environment (60, 61). During refrigeration, counts declined gradually from 11.50 log CFU on day 2 to 11.27 log CFU/mL on day 21. This decline is due to acid stress, nutrient depletion, and oxygen exposure, which are vital factors in probiotic survival in fermented foods (62). Although the cell count declines, it remains above 10^8^ CFU/mL, exceeding the FAO/WHO recommended minimum threshold (10^6^ CFU/mL) for conferring health benefits.

**Figure 14:**
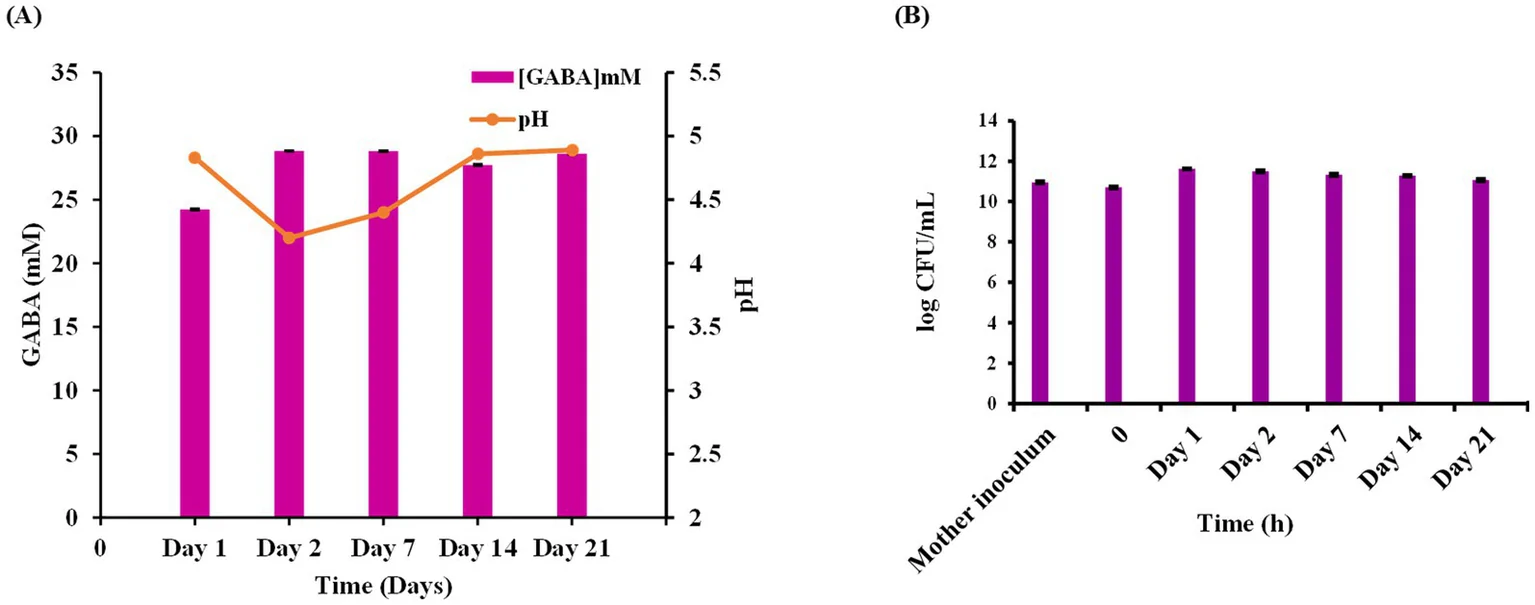
**(A)** GABA-enriched yogurt stability test during a storage period of 21 days. The error bars represent mean ± SD of three independent experiments. **(B)** Cell viability of *E. faecalis* RIT BM_S15-3_ after fermentation in almond yogurt during a storage period of 21 days. The error bars represent mean ± SD of three independent experiments.

#### 3.8.6 Storage stability of GABA in almond yogurt

The GABA-enriched almond yogurt showed excellent storage stability during refrigerated storage, with GABA concentrations remaining unchanged throughout the 21 days. The maximum GABA concentration (29 mM) was observed on day 2 (Figure 14A) and was subsequently maintained until day 21, demonstrating that the synthesized GABA remained highly stable within the almond yogurt matrix. This type of stability is a desirable characteristic for functional foods, since it assures that customers receive the targeted bioactive dose throughout the shelf-life of the product.

The observed stability can be attributed to the protective effect of the almond yogurt matrix and the metabolic characteristics of GABA-producing *E. faecalis* RIT BM_S15-3_. GABA accumulated during fermentation through the GAD pathway was highly retained throughout the 21-day refrigerated storage period. This is consistent with previous studies on GABA-enriched yogurt, where refrigeration effectively suppresses bacterial metabolic activity, limiting further synthesis and enzymatic degradation (18). The structural composition of the almond yogurt matrix may have contributed to this stability. Proteins, polysaccharides, and peptides inherent to the fermented system are known to form a protective microenvironment that reduces biochemical degradation of bioactive metabolites (63, 68). This is supported by recent reviews on GABA-enriched dairy products, which report that *in situ* fermentation using GABA-producing LAB yields superior GABA stability during storage compared to direct fortification strategies. This effect can be attributed to the metabolite’s association with a biologically active and structurally cohesive food matrix (64, 65). pH remained stable at 4.8 ± 0.005 throughout storage, suggesting negligible post-acidification and a metabolically balanced microbial community. Excessive post-acidification has been shown to compromise bacterial viability and alter the stability of functional metabolites (66). The sustained pH shown here is aligned with the results from comparable GABA-enriched fermented dairy systems, where stable pH during refrigerated storage was correlated with LAB viability and preservation of bioactive compounds (67).

## 4 Conclusion

This study demonstrated the potential of human breast milk-derived *E.faecalis* RIT BM_S15-3_ as a GABA-producing probiotic candidate for the development of an almond-based fermented yogurt. Fermentation of almond milk with *E.faecalis* RIT BM_S15-3_ resulted in effective acidification, high viable counts, GABA enrichment, and improved functional properties, including antioxidant activity and gastrointestinal tolerance. Optimization of fermentation conditions identified 48 h fermentation at 37 °C with 3 g/L L-MSG and 10% inoculum as optimal, yielding a maximum GABA concentration of 29 mM in the confirmatory experiment. The fermented almond yogurt also maintained GABA stability during 21 days of storage and demonstrated acceptable sensory characteristics, supporting its potential as a naturally GABA-enriched non-dairy fermented food.

However, the present study has a few important translational limitations. The functional and psychobiotic-related evaluations were primarily based on *in vitro* assays, and no *in vivo* animal behavioral or neurobiological studies were performed to confirm the proposed stress-relieving, anxiolytic, or neuroprotective effects of the GABA-enriched almond yogurt. Thus, the current findings should be considered proof of concept for product development rather than definitive evidence of psychobiotic efficacy.

Future studies should focus on rodent anxiety and stress models to evaluate behavioral and neurobiological responses and investigate potential gut-brain-axis-mediated effects. Pilot-scale fermentation and process optimization will be necessary to assess reproducibility, scalability, and industrial feasibility. Moreover, sensory and formulation optimization should be undertaken to improve flavor, texture, and overall acceptability and to narrow the sensory acceptance gap between almond yogurt and commercial dairy yogurt. These studies, followed by appropriately designed human intervention trials, will be essential to establish the safety, efficacy, and translational potential of GABA-enriched almond yogurt.

## Data Availability

The data analyzed in this study is available in the NCBI repository (https://www.ncbi.nlm.nih.gov) under accession PRJNA1480835.
