# Correction: Production and evaluation of a novel GABA-producing almond yogurt: dietary modulation of stress

**DOI:** 10.3389/fvets.2026.2019068

**Published:** 2026-10-28

**Authors:** Murali Anagha, Roshni Ramachandran, Gangaraju Divyashri, T. P. Krishna Murthy

**Affiliations:** 1 Department of Biotechnology, M S Ramaiah Institute of Technology, Bengaluru, Karnataka, India; 2 Bioproducts Research, Iom Bioworks Pvt. Ltd, Centre for Cellular and Molecular Platforms (C-CAMP), Bengaluru, Karnataka, India

**Keywords:** almond yogurt, *Enterococcus faecalis* RIT BMS15-3, functional food, gamma-aminobutyric acid (GABA), psychobiotics

There was a mistake in Figure 13 as published.

The figure displayed in the published version is the ABTS inhibition graph, whereas the accompanying caption corresponds to the sensory evaluation results of almond yogurt, peanut yogurt, and dairy yogurt.

The corrected Figure 13 appears below.

**Figure 13:**
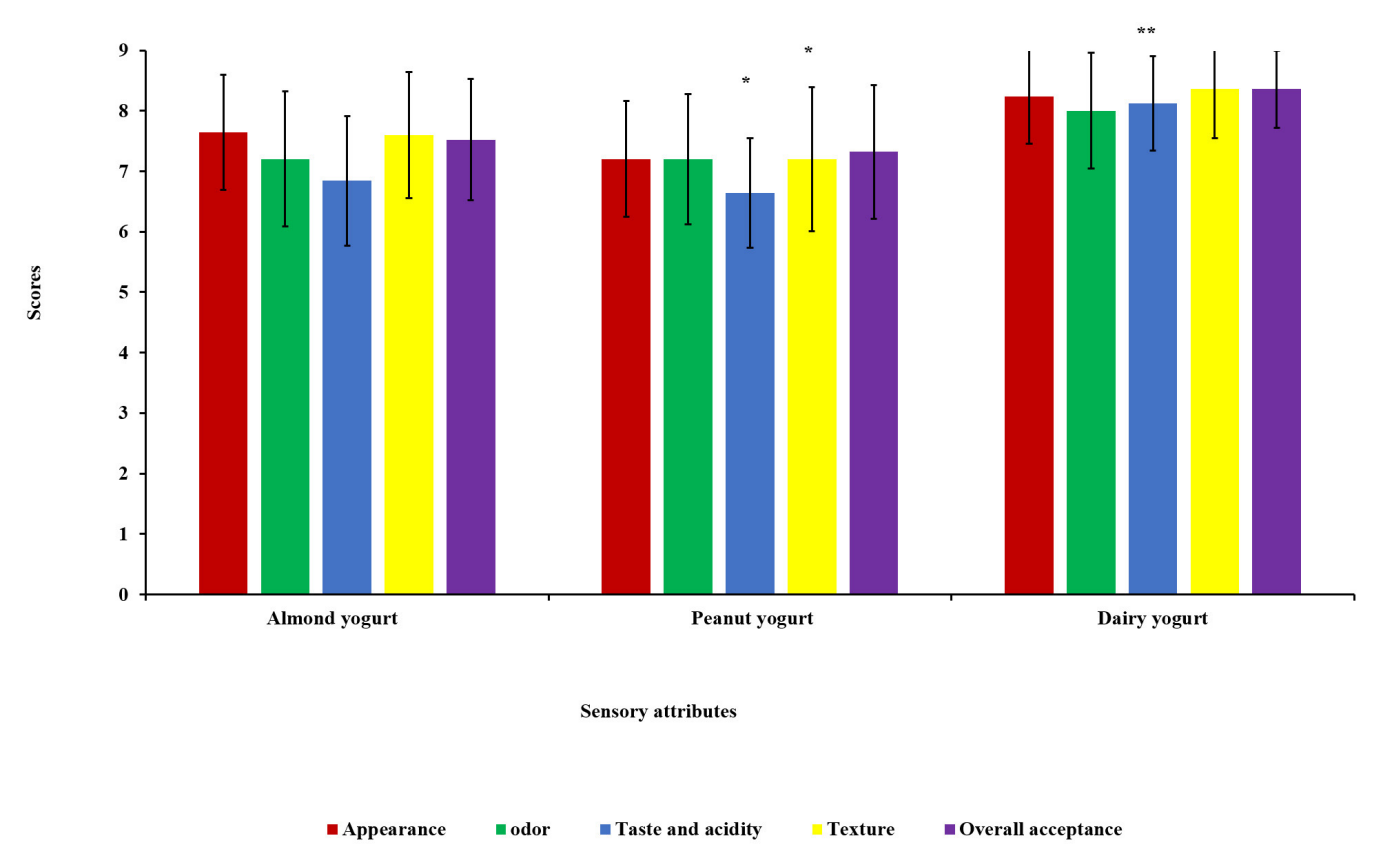
Sensory evaluation scores of almond yogurt, peanut yogurt, and dairy yogurt for appearance, odor, taste and acidity, texture, and overall acceptance. Data are represented as mean ± SD. Pearson correlation analysis was performed to assess the sensory attributes. Significant correlations were observed for taste and acidity of peanut yogurt (*p < 0.05) and taste and acidity of dairy yogurt (**p < 0.01).

There was a mistake in the caption of **Figure 1** as published. Gastrointestinal tolerance of *E. faecalis* RIT BMS15-3 in almond yogurt during storage. The error bars represent the mean ± SD of three independent experiments in.

The corrected caption of **Figure1** appears below.

**Figure 1**. Gastrointestinal tolerance of *E. faecalis* RIT BM_S15 − 3_ in almond yogurt during storage. The error bars represent the mean ± SD of three independent experiments.

The original version of this article has been updated.

