# Supplementary material for: Production and evaluation of a novel GABA-producing almond yogurt: dietary modulation of stress
Source: Front Vet Sci. 2026 Sep 21;13:1949462. doi: 10.3389/fvets.2026.1949462 (PMC13637514; doi:10.3389/fvets.2026.1949462)
Supplement: Supplementary file 1 [file Table_1.DOCX]

| **Antibiotic** | **Result** | **Interpretation** |
| --- | --- | --- |
| **Azithromycin** | **Resistant** | **Resisitance detected** |
| **Vancomycin** | **Sensitive** | **-** |
| **Ciprofloxacin** | **Resistant** | **Resisitance detected** |
| **Amikacin** | **Sensitive** | **-** |
| **Linezolid** | **Sensitive** | **-** |
| **Tetracycline** | **Sensitive** | **-** |
| **Rifamycin** | **Sensitive** | **-** |
| **Erythromycin** | **Resistant** | **Resisitance detected** |
| **Penicillin-G** | **Resistant** | **Resisitance detected** |
| **Ampicillin** | **Sensitive** | **-** |
| **Acid tolerance** | **11.435 log CFU/mL after 1.5 h of exposure** | **-** |
| **Bile tolerance** | **11.86 log CFU/mL at 0.3% bile salts** | **-** |
| **Hemolytic activity** | **Negative** | **No detectable hemolytic phenotype** |
| **Gelatinase activity** | **Negative** | **No detectable gelatinase activity** |
| **Whole-Genome Sequencing** |  |  |
| ***Van A*** | **Not detected** | **No acquired vancomycin resistance** |
| ***Van B*** | **Not detected** | **No acquired vancomycin resistance** |
| ***esp*** | **Not detected** | **No virulence surface protein** |
| ***cyl*** | **Not detected** | **No cytolysin-associated virulence factor** |
| ***gelE*** | **Detected** | **Gelatinase involved in extracellular proteolysis** |
| ***hyl*** | **Not detected** | **No tissue invasion** |
| ***fsr A/fsr B/fsr C*** | **Not detected** | **No quorum sensing** |

**Supplementary Table S1. Previously reported antimicrobial susceptibility profile of *E. faecalis* RIT BM_S15-3_ , acid and bile tolerance, and safety analysis**
